# Switching Rat Resident Macrophages from M1 to M2 Phenotype by Iba1 Silencing Has Analgesic Effects in SNL-Induced Neuropathic Pain

**DOI:** 10.3390/ijms242115831

**Published:** 2023-10-31

**Authors:** Roxana-Olimpia Gheorghe, Andreea Violeta Grosu, Melania Magercu, Mihail-Sebastian Ghenghea, Cristina Elena Zbarcea, Alexandra Tanase, Simona Negres, Alexandru Filippi, Gabriela Chiritoiu, Mihaela Gherghiceanu, Sorina Dinescu, Gisela Gaina, Damir Sapunar, Violeta Ristoiu

**Affiliations:** 1 Department of Anatomy, Animal Physiology and Biophysics, Faculty of Biology, University of Bucharest, 91-95 Splaiul Independentei, District 5, 050095 Bucharest, Romania; roxana.gheorghe@bio.unibuc.ro (R.-O.G.);; 2 Department of Pharmacology and Clinical Pharmacy, Faculty of Pharmacy, University of Medicine and Pharmacy “Carol Davila”, 6 Traian Vuia Street, District 2, 02095 Bucharest, Romania; 3 Department of Biophysics, University of Medicine and Pharmacy “Carol Davila”, 8 Eroilor Sanitari Blvd., 050474 Bucharest, Romania; 4 Department of Molecular Cell Biology, Institute of Biochemistry, Romanian Academy, 2996 Splaiul Independentei 296, District 6, 060031 Bucharest, Romania; 5 Ultrastructural Pathology and Bioimaging Laboratory, Victor Babeș National Institute of Pathology Bucharest, 99-101 Splaiul Independentei, District 5, 050096 Bucharest, Romania; 6 Department of Biochemistry and Molecular Biology, University of Bucharest, 91-95 Splaiul Independentei, District 5, 050095 Bucharest, Romania; 7 Department of Anatomy, Histology and Embryology, University of Split School of Medicine, Šoltanska 2, 21000 Split, Croatia

**Keywords:** Iba1 protein, macrophages, siRNA, SNL-induced neuropathic pain, M1 and M2 phenotypes

## Abstract

Resident macrophages from dorsal root ganglia are important for the development of traumatic-induced neuropathic pain. In the first 5–7 days after a traumatic sciatic nerve injury (i.e., spinal nerve ligation (SNL), spared nerve injury (SNI), sciatic nerve transection or sciatic nerve ligation and transection), Ionized binding adapter protein 1 (Iba1) (+) resident macrophages cluster around dorsal root ganglia neurons, possibly contributing to nerve injury-induced hypersensitivity. Since infiltrating macrophages gradually recruited to the lesion site peak at about 7 days, the first few days post-lesion offer a window of opportunity when the contribution of Iba1 (+) resident macrophages to neuropathic pain pathogenesis could be investigated. Iba1 is an actin cross-linking cytoskeleton protein, specifically located only in macrophages and microglia. In this study, we explored the contribution of rat Iba1 (+) macrophages in SNL-induced neuropathic pain by using intra-ganglionic injections of naked Iba1-siRNA, delivered at the time the lesion occurred. The results show that 5 days after Iba1 silencing, Iba1 (+) resident macrophages are switched from an M1 (pro-inflammatory) phenotype to an M2 (anti-inflammatory) phenotype, which was confirmed by a significant decrease of M1 markers (CD32 and CD86), a significant increase of M2 markers (CD163 and Arginase-1), a reduced secretion of pro-inflammatory cytokines (IL-6, TNF-α and IL-1β) and an increased release of pro-regenerative factors (BDNF, NGF and NT-3) which initiated the regrowth of adult DRG neurites and reduced SNL-induced neuropathic pain. Our data show for the first time, that it is possible to induce macrophages towards an anti-inflammatory phenotype by interacting with their cytoskeleton.

## 1. Introduction

Traumatic events result in peripheral nerve injuries, which are frequently accompanied by painful neuropathic pain that develops as a result of neuronal dysfunction [1,2,3] and numerous neuro-immune interactions [4,5,6,7]. Among them, the bidirectional communication between activated macrophages and traumatically lesioned peripheral nerves, which starts very quickly after the lesion occurs, is very important [8,9,10]. This crosstalk takes place at the level of peripheral nerves and sensory ganglia and involves resident and infiltrating macrophages [10,11], but there is an ongoing debate on which macrophages contribute more.

Some studies suggest that macrophages infiltrating the nerve injury are more important. Clodronate depletion of infiltrating monocytes in a mice partial sciatic nerve ligation model significantly attenuated macrophage infiltration in the injured nerve and reduced allodynia [12,13], while the chemogenic depletion of macrophages at the nerve injury site reduced the SNI-induced mechanical hyperalgesia in macrophage Fas-induced apoptosis (MAFIA) transgenic mice [14]. Since dorsal root ganglia (DRG) macrophages were spared, the authors concluded that peripheral macrophages were the critical contributors to pain [14].

Other studies suggest that macrophages at the DRG level (more commonly investigated) are more important. MAFIA mice with chemogenically depleted DRG resident macrophages developed mechanical allodynia 7 days after SNI, compared with a 24 h timeframe in non-treated animals, and showed no sign of infiltrating macrophages [15]. Since only the DRG macrophages were affected, the authors concluded that DRG macrophages were critical contributors to pain [15]. Similarly, clodronate-treated mice that lost resident DRG macrophages post-SNI showed reduced mechanical allodynia and cold hypersensitivity [16].

Resident DRG macrophages are a distinct population [17,18,19] that is also identified by specific markers: resident macrophages generally express Iba1 and ED2/CD163, while infiltrating macrophages express ED1/CD68, OX-42/CD11b, F4/80, MAC1 and MHC-II [16,20,21,22]; a certain degree of Iba1 and ED1 overlapping was described 3 days after sciatic nerve transection [23].

After a nerve injury, resident Iba1 (+) macrophages form ring-like clusters around neuronal bodies, i.e., 5 days after SNL [24], 7 days after SNI [25], 7 days after sciatic nerve transection [26] or 7 days after sciatic nerve ligation and transection [27]. Infiltrating macrophages are recruited 2–3 days after the lesion occurs, peaking at 7 days [28]: i.e., ED1 (+) macrophages infiltrated 5 days after SNL form distinct clusters from Iba1 (+) clusters around DRG neurons [24], they are still present 7 and 14 days after SNL, but ED1 and Iba1 overlapping has not been investigated [29]. In the brain, Iba1 (−) circulating monocytes become Iba1 (+) only after they enter the brain and mature [30,31], which is a possible process for DRG that has not yet been investigated.

Based on the data above, we considered that the first days after the occurrence of a traumatic nerve lesion are a window of opportunity, when the contribution of Iba1 (+) resident macrophages to neuropathic pain pathogenesis could be investigated. In this study, we explored their contribution to SNL-induced neuropathic pain by interfering with Iba1, a cytoskeleton protein specific to macrophages and microglia [32,33,34,35,36]. Previously, we have shown that silencing Iba1 in BV2 microglia significantly affected their migration, proliferation and phagocytosis [37], suggesting that similar effects could be obtained in resident macrophages, as well. We hypothesized that local administration of the Iba1-siRNA at the L5 DRG level immediately after the lesion occurs will interfere with the clustering of activated Iba1 (+) macrophages around neurons, will reduce their pro-nociceptive actions on DRG neurons [8,15] and consequently, will reduce pain. The results show that 5 days after the lesion occurs, Iba1 silencing induced an M1 to M2 switch in the DRG macrophages, accompanied by a reduced secretion of pro-inflammatory cytokines, an increased secretion of pro-regenerative factors that stimulated regrowth of adult DRG neurites and reduced SNL-induced neuropathic pain.

## 2. Results

### 2.1. SNL-Induced Clusters of Iba1 (+) Macrophages around DRG Neurons Are Maintained Even after Iba1 Silencing

**Figure 1:**
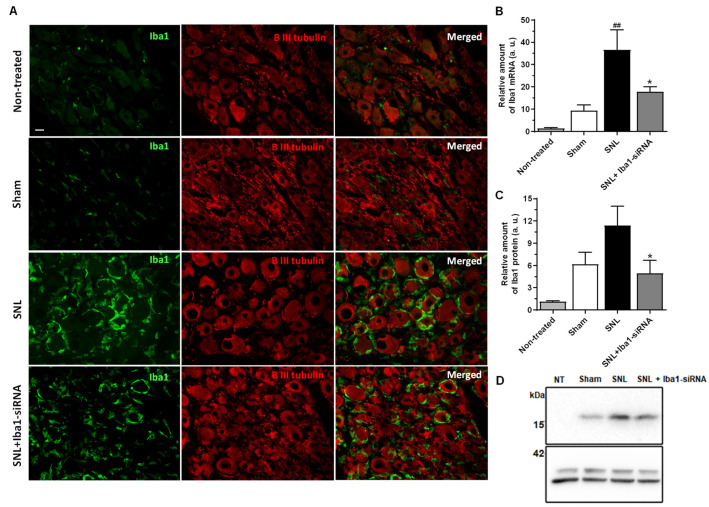
Iba1(+) rings of macrophages around DRG neurons are maintained after Iba1 silencing. (**A**) Double immunostaining for Iba1 and βIII-tubulin (a non-specific marker for neurons), indicating that ring-like clusters of Iba1 (+) macrophages around most of the neurons in L5 DRG, 5 days after SNL, are somewhat disorganized after intra-ganglionic delivery of Iba1-siRNA (scale bar: 20 μm). (**B**) Validation of Iba1 silencing by qRT-PCR. Iba1 mRNA level was significantly up-regulated after SNL, compared to the sham group (^##^ *p* < 0.01), it was significantly down-regulated after injection of Iba1-siRNA (* *p* < 0.05), but it was not different compared to the sham condition (*p* > 0.05). (**C**) Bar graph representing mean concentration of Iba1 protein, quantified from individual L5 DRG. Iba1-siRNA induced a significant reduction of Iba1 protein, in the same range as for mRNA silencing (* *p* < 0.05 vs. SNL group), to a level very close to the sham condition (*p* > 0.05). (**D**) Representative blot image of Iba1 expression in different experimental conditions. A band corresponding to Iba1 was detected at the predicted molecular weight (~17 KDa). NT: non-treated; SNL: spinal nerve ligation.

To explore whether Iba1 silencing alters the SNL-induced clusters of Iba1 (+) macrophages around neurons, we examined sections of DRG from all conditions stained with anti-Iba1 antibody. In non-treated and sham conditions, Iba1 (+) macrophages were scattered between neurons (Figure 1A, rows 1–2), as we have previously shown [24], while 5 days after SNL they formed tight peri-neuronal rings around most of the neurons (Figure 1A, row 3), with macrophages in close contact with one another inside the rings and between the rings. After Iba1-siRNA injection, the rings were still present, although somewhat disorganized, with macrophages not as tightly organized inside the ring as after SNL and with the Iba1 expression slightly reduced in intensity (Figure 1A, row 4).

The efficacy of Iba1 silencing was first confirmed by qRT-PCR (Figure 1B). The results showed that SNL surgery induced a significant increase of Iba1 mRNA compared to sham conditions (relative amount of mRNA after sham surgery = 9.41 ± 2.49, *n* = 9, and after SNL = 36.56 ± 9.04, *n* = 9, ~74% increase, *p* = 0.001), which was significantly reduced after the injection with 400 nM Iba1-siRNA (relative amount of mRNA = 17.81 ± 2.26, *n* = 9, *p* = 0.029), indicating a ~50% silencing efficacy. The Iba1 mRNA level after treatment was not significantly different compared to the sham condition (*p* = 0.682). This efficacy was also confirmed at the protein level by Western Blot (Figure 1C,D). The results showed that SNL surgery induced an increase in Iba1 protein compared to the sham condition, although not significantly (relative amount of Iba1 protein after sham surgery = 6.16 ± 1.61, *n* = 8, and after SNL = 11.38 ± 2.59, *n* = 8, ~46% increase, *p* = 0.124), which was significantly reduced after the injection with 400 nM Iba1-siRNA (relative amount of protein = 4.94 ± 1.75, *n* = 8, *p* = 0.041), indicating a ~56% silencing efficacy. The Iba1 protein level after treatment was not significantly different compared to the sham condition (*p* = 0.999). The increase in both mRNA and Iba1 protein levels in the sham condition, compared to the non-treated condition, was expected, considering that sham surgery involved nerve manipulation and random injections of 0.2× phosphate-buffered saline (PBS) or 400 nM scramble-siRNA into the L5 DRG and that macrophages react very quickly to any injury in the surrounding environment [8,38]. Nevertheless, the increase was insignificant (relative amount of mRNA in the non-treated condition = 1.36 ± 0.38, *n* = 9, *p* = 0.988, and relative amount of Iba1 protein in the non-treated condition = 1.12 ± 0.12, *n* = 9, *p* = 0.167).

To confirm the identity of Iba1 (+) macrophages as a distinct population from ED1 (+) macrophages and satellite cells, we refer to previously published data [24], presented here as supplemental data with copyright from Elsevier, 2022. Specifically, we have shown that 5 days after SNL, the cells around DRG neurons can be Iba1 (+), ED1 (+) or MHC-II (+) (Supplemental Figure S6A,B). The ED1 expression did not overlap with Iba1 (Supplemental Figure S6(Ac)), which suggests that the ED1 (+) cells form a separate population of macrophages inside the DRG. Meanwhile, MHC-II (+) expression was more complex, with some Iba1 (+) cells expressing MHC-II marker, as well (Supplemental Figure S6(Bc)). Additionally, we have shown that Iba1 (+) macrophages are also distinct from satellite cells (Supplemental Figure S6(Ca–f)) and were apparently infiltrated under the satellite cells, closer to the neuronal body, 5 days after SNL (Supplemental Figure S6(Cc,f)).

**Figure 2:**
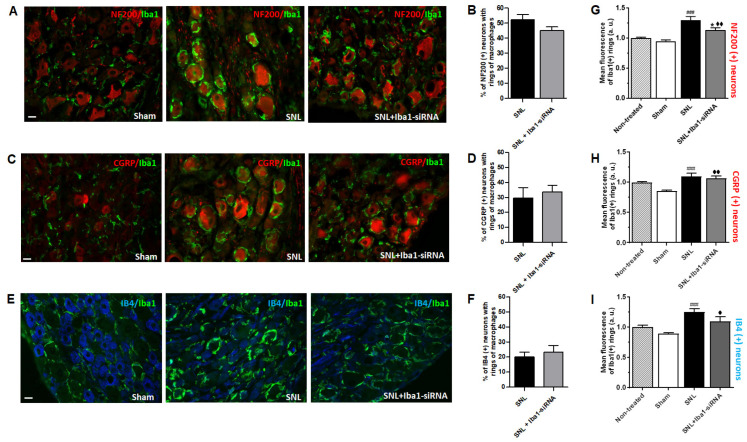
Although all types of DRG neurons were surrounded by Iba1 (+) macrophages, only the rings around large NF200 (+) DRG neurons became looser after Iba1 silencing. (**A**,**C**,**E**) Double immunostaining for Iba1 and NF200, GCRP and IB4 (+) L5 DRG neurons, indicating rings of Iba1 (+) macrophages around them, tightly organized 5 days after SNL or somewhat disorganized after treatment with Iba1-siRNA (scale bar: 20 μm). (**B**,**D**,**F**) Bar graphs showing that the percentage of Iba1 (+) rings around NF200, GCRP and IB4 (+) neurons was not altered by Iba1 silencing (*p* > 0.05). (**G**,**H**,**I**) Bar graphs of mean fluorescence intensity of Iba1 (+) macrophages, indicating that after SNL, all neurons had rings around them (^###^ *p* < 0.001). After Iba1 silencing, only the Iba1 (+) rings around NF200 (+) became looser (* *p* < 0.05), but they were still denser compared to the sham condition (^◆◆^ *p* < 0.01), while for CGRP (+) and IB4 (+) neurons there was no significant change (*p* > 0.05) compared to SNL. However, the Iba1 (+) rings were still denser than in the sham condition (^◆◆^ *p* < 0.01 and ^◆^ *p* < 0.05, respectively).

To confirm if the rings of macrophages were indeed disorganized after Iba1 silencing, we explored their characteristics in detail. First of all, since most of the neurons were surrounded by tight rings of macrophages, we investigated if Iba1 (+) rings develop preferentially around a type of neurons, i.e., NF200 (+) (large), CGRP (+) (small, peptidergic) or IB4 (+) (small, non-peptidergic). The results showed the following preference after SNL (percentage indicates neurons with Iba1 (+) rings out of the total number of neurons of a certain type, pooled from 3 animals): NF200 (+) (52.18%, *n* = 608) > CGRP (+) (29.68%, *n* = 250) > IB4 (+) (20.20%, *n* = 307), which did not change significantly after Iba1 silencing: NF200 (+) (45.13%, *n* = 393, *t*-test, *p* = 0.081) (Figure 2A,B) > CGRP (+) (33.82%, *n* = 393, *t*-test, *p* = 0.594) (Figure 2C,D) > IB4 (+) (23.24%, *n* = 142, *t*-test, *p* = 0.578) (Figure 2E,F).

**Figure 3:**
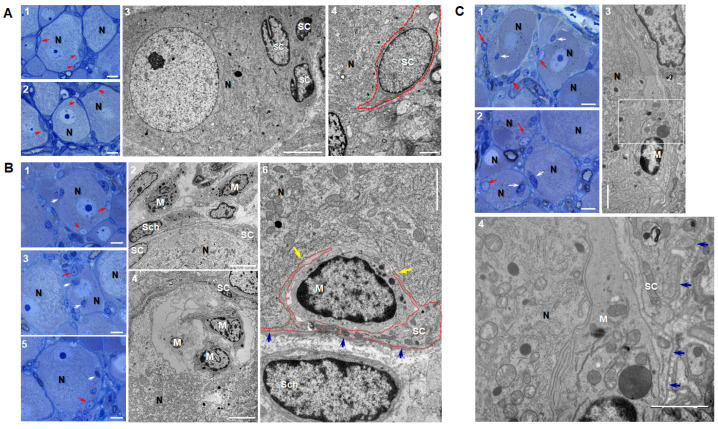
(**A**) Light and electron microscopy of normal L5 dorsal root ganglia. Light microscopy (1) and (2) represent normal DRG structure on toluidine blue stain sections (diameter = 1 μm). Red arrows indicate satellite cells surrounding DRG neurons (scale bar = 10 μm). Electron microscopy images (3) and (4) show the detailed, ultrathin structure of the border between the neuron and the surrounding satellite cells. In (4), the dashed red line highlights the edges of the satellite cell near the neuron. Scale bars: in (3) = 5 μm and in (4) = 2 μm. N—neuron; SC—satellite cell. (**B**) Light (1, 3 and 5) and electron microscopy (2, 4 and 6) of L5 dorsal root ganglia 5 days after SNL surgery. (1) and (2) Macrophages form peri-neuronal rings around neurons, visible on thin sections (1, white arrows—macrophage, red arrows—satellite cells; scale bar = 10 μm) or on ultrathin sections (2, scale bar = 5 μm). (3) and (4) Macrophages are engulfed in the neuronal cytoplasm, visible on thin sections (3, white arrows—macrophages; red arrows—satellite cells; scale bar = 10 μm) or on ultrathin sections (4, scale bar = 5 μm). In (4), a vacuole containing three macrophages is clearly delimited inside the cytoplasm and from the satellite cells sheath. (5) and (6) Macrophages are surrounded by a “*pocket*” formed by a satellite cell’s extensions in the space between the satellite cell and the neuronal body, visible on thin sections (5, white arrows—macrophages; red arrows—satellite cells; scale bar = 10 μm) or on ultrathin sections (6, the dashed red line highlights the edges of the satellite cell around the macrophages, yellow arrows—extensions of the satellite cells, blue arrows—basal membrane of the satellite cell, scale bar = 2 μm). N—neuron, M—macrophages, SC—satellite cell, Sch—Schwann cell. (**C**) Intra-ganglionic delivery of Iba1-siRNA partially reduced the macrophages-neuronal’ interactions. The peri-neuronal ring of macrophages is still present around neurons after Iba1-siRNA delivery (1), and some macrophages seem to be located under the satellite cell’s sheath, visible in (1) and (2) (light microscopy on thin sections, white arrows—macrophages, red arrows—satellite cells; scale bar = 10 μm). (3) Electron microscopy image shows a macrophage between the neuron and the satellite cell’s sheath (scale bar = 5 μm). (4) High-magnification image of the selected area in (3). The disposition of the macrophage between the satellite cell and the neuron is clearly visible. Blue arrows indicate the basal membrane of the satellite cell (scale bar = 2 μm). N—neuron, M—macrophages, SC—satellite cell.

To confirm the ring tightness around neurons, we quantified the ring area of Iba1 (+) macrophages around all types of DRG neurons. The results showed that for all types of neurons, after SNL, the rings were denser, with significantly more Iba1 (+) macrophages on and around the neurons (which is the equivalent of increased mean fluorescence of the rings) compared to the sham condition (mean fluorescence of Iba1 (+) rings for: (1) NF200 (+) neurons after sham surgery = 0.94 ± 0.02, *n* = 3, and after SNL = 1.29 ± 0.06, *n* = 3, *p* < 0.001, Figure 2G; (2) CGRP (+) neurons after sham surgery = 0.85 ± 0.01, *n* = 3, and after SNL = 1.09 ± 0.05, *n* = 3, *p* < 0.001, Figure 2H; (3) IB4 (+) neurons after sham surgery = 0.88 ± 0.01, *n* = 3, and after SNL = 1.24 ± 0.05, *n* = 3, *p* < 0.001, Figure 2I). After Iba1 silencing, the rings became significantly looser, although not completely disorganized, only for NF200 (+) neurons (mean fluorescence of Iba1 (+) rings after SNL + Iba1-siRNA = 1.13 ± 0.03, *n* = 3, *p* = 0.020, Figure 2A,G). Meanwhile, for CGRP (+) and IB4 (+) neurons there was no significant change (mean fluorescence of Iba1 (+) rings after SNL + Iba1-siRNA for CGRP (+) neurons = 1.06 ± 0.03, *n* = 3, *p* = 0.999, Figure 2C,H; for IB4 (+) neurons = 1.09 ± 0.07, *n* = 3, *p* = 0.118, Figure 2E,I). The Iba1 (+) rings were still denser after Iba1 silencing compared to sham conditions for all types of DRG neurons (for NF200 (+) neurons, *p* = 0.004, Figure 2G; for CGRP (+) neurons, *p* = 0.001, Figure 2H and for IB4 (+) neurons, *p* = 0.012, Figure 2I).

Furthermore, we performed an electron microscopy study to explore the structure of the rings, and determine whether the macrophages are actually in close contact with one another inside the rings and between the rings. In a previous study, after LPS treatment, satellite glial cells showed elongated cytoplasmic processes projecting into the connective tissue space, often towards other cells, finally establishing new gap junctions [39]. Our hypothesis was that Iba1 (+) macrophages could have similar cytoplasmic projections within the peri-neuronal ring, possibly some gap junctions between them, and perhaps some extensions towards neurons or satellite cells, as well.

The results showed that in normal conditions, the DRG neurons were closely surrounded by satellite cells (Figure 3A(1–4)). The differential ultrastructural diagnostic between satellite cells and macrophages was made mainly based on the cell’s shape, nuclear morphology, the abundance of cellular organelles, cellular extensions and the presence or absence of external basal lamina. Five days after SNL surgery, we noticed three different behaviors of macrophages: (1) they formed a peri-neuronal ring around neurons (Figure 3B(1,2)); (2) they were completely engulfed in the cytoplasm of the neurons (Figure 3B(3,4)) and (3) they infiltrated between satellite cells and the neuronal body, in what we called “*in-pocket*” clustering (Figure 3B(5,6)). The peri-neuronal ring consisted of macrophages clustered near the neuron, outside the satellite cells sheath, which did not lead to contacts between each other or with the satellite cells (Figure 3B(2)). The vacuole containing engulfed macrophages (Figure 3B(4)), had a clear membrane at the border and was not associated with the disruption of the satellite cells sheath. Five days after the SNL surgery combined with Iba1-siRNA injection, the peri-neuronal distribution of macrophages was still visible (Figure 3C(1)), and the “*in-pocket*” clustering was replaced by “*under the sheath*” clustering, where macrophages were clearly disposed under the satellite cells sheath and had no satellite cell extensions around them (Figure 3C(2–4)). The intracytoplasmic macrophages were no longer visible.

The results in this section confirm that despite one nucleotide mismatch between Iba1-siRNA and rat Iba1 mRNA, it was possible to induce ≥50% Iba1 silencing at both mRNA and protein levels in rat DRG, but this was not enough to prevent Iba1 (+) rings from forming around all DRG neurons. Surprisingly, the rings formed around NF200 (+), CGRP (+) or IB4 (+) DRG neurons were of different “strengths”, since Iba1 silencing significantly disorganized only the rings formed around NF200 (+) neurons, with possible functional consequences.

### 2.2. Intra-Ganglionic Delivery of Iba1-siRNA Significantly Reduced the SNL-Induced Neuropathic Pain, without Reducing Neuronal Excitability

**Figure 4:**
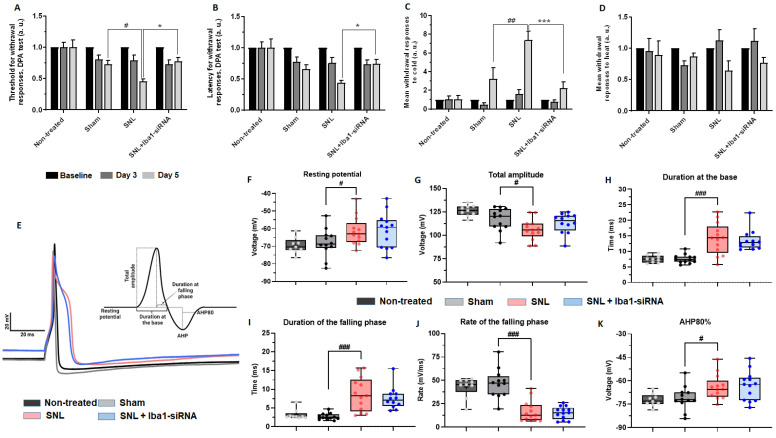
Intra-ganglionic delivery of Iba1-siRNA reduced SNL-induced hyperalgesia, but it did not reduced neuronal excitability. (**A**) The significant decrease of threshold for withdrawal responses in a DPA test after SNL surgery, compared to the sham group (^#^ *p* < 0.05), was significantly attenuated after injection with 400 nM Iba1-siRNA (* *p* < 0.05). (**B**) In contrast, the reduction of latency for withdrawal responses in a DPA test after SNL surgery, compared to the sham group, was not significant (*p* > 0.05), but it was considerably attenuated after injection with 400 nM Iba1-siRNA (* *p* < 0.05). (**C**) The increased mean number of withdrawal responses to acetone after SNL surgery (^##^ *p* < 0.01) was significantly reduced after injection with 400 nM Iba1-siRNA (*** *p* < 0.001). (**D**) Sensitivity to heat was not affected after SNL. (**E**) Representative traces of action potentials in different experimental conditions. Inset represents how different parameters presented in the graphs on the right were measured (created with BioRender.com (accessed on 25 October 2023). Bar graphs showing significant effects on the mean values for: (**F**) resting potentials (^#^ *p* < 0.05), (**G**) total amplitude (^#^ *p* < 0.05), (**H**) duration at the base (^###^ *p* < 0.001), (**I**) duration of the falling phase (^###^ *p* < 0.001), (**J**) rate of the falling phase (^###^ *p* < 0.001) and (**K**) amplitude of AHP at 80% recovery (^#^ *p* < 0.05), which were not attenuated by the Iba1-siRNA injection (*p* > 0.05). SNL—spinal nerve ligation, DPA—dynamic plantar aesthesiometer, AHP—afterhyperpolarization.

To investigate if Iba1 silencing has functional consequences, the pain sensitivity to mechanical and thermal stimuli was subsequently evaluated 3 and 5 days post SNL, to monitor the SNL-induced neuropathic pain development and Iba1 silencing effects on it (Figure 4A–D and Supplemental Table S3 with one-way ANOVA repeated measures). Since day 5 was our end-point measurement, only these values were used for further analysis.

The results showed that 5 days after SNL surgery, the animals developed mechanical allodynia. The threshold for withdrawal in the dynamic plantar aesthesiometer (DPA) test was significantly lower compared to the sham condition (mean threshold for the sham group = 0.72 ± 0.06, *n* = 11, and after SNL = 0.45 ± 0.03, *n* = 9, ~38% decrease, *p* = 0.036, Figure 4A). However, the latency for withdrawal did not diminish significantly, with the decrease percentage proving to be quite similar to the threshold for withdrawal (mean latency for the sham group = 0.65 ± 0.07, *n* = 11, and after SNL = 0.43 ± 0.04, *n* = 9, ~34% decrease, *p* = 0.214, Figure 4B). The lack of significance for the latency of withdrawal in the DPA test, in contrast to what was previously described [40,41,42], is due to the fact that in our pain model, sham surgery involved nerve manipulation and random injections of 0.2× PBS or 400 nM scramble-siRNA into the L5 DRG. This consequently induced a significant decrease in the latency value for the sham condition compared to the non-treated condition (mean latency for the non-treated group = 1.00 ± 0.14, *n* = 8, *p* = 0.028). After SNL surgery, there was an additional latency decrease, but it was not significant compared to the sham condition. On the other hand, the threshold in the sham condition was not significantly different compared to the non-treated condition (mean threshold for the non-treated group = 1.00 ± 0.11, *n* = 8, *p* = 0.062), which allowed a further decrease after SNL surgery to a significant level compared to the sham condition (*p* = 0.036). Our results for the sham condition confirm the data in the literature that intra-ganglionic injection of saline (in our case, PBS or scramble-siRNA formulated in PBS) can induce a local inflammatory response [38], which is not accompanied, however, by the same marked hyperalgesia as the one induced by SNL surgery. After Iba1-siRNA injection, the reduction of both threshold and latency was significantly attenuated: mean threshold after siRNA treatment = 0.77 ± 0.06, *n* = 12, *p* = 0.010, Figure 4A; mean latency after siRNA treatment = 0.74 ± 0.06, *n* = 12, *p* = 0.035, Figure 4B. There was no difference for threshold and latency in the DPA test between Iba1-siRNA injection and sham conditions (*p* = 0.999 for both), indicating that after Iba1 silencing, the mechanical sensitivity is restored close to the sham level.

SNL-induced thermal hypersensitivity was evaluated using stimulation with acetone for cold and stimulation on a hot plate for heat. The results showed that after SNL surgery, the sensitivity to acetone significantly increased compared to sham conditions (mean number of responses for the sham group = 3.2 ± 1.26, *n* = 11, and after SNL = 7.36 ± 0.94, *n* = 9, ~56% increase, *p* = 0.007, Figure 4C), as previously described [40,42]. After Iba1-siRNA injection, the sensitivity significantly reduced compared to the SNL group (mean number of responses after siRNA treatment = 2.24 ± 0.67, *n* = 12, ~69% decrease, *p* < 0.001, Figure 4C), being restored to a sham level sensitivity, compared to which there was no significant difference (*p* = 0.999, Figure 4C). After SNL surgery, the sensitivity to heat was not different in this group compared to the other groups (see Supplemental Table S3 for numerical values comparing sham vs. SNL, *p* = 0.590; SNL vs. Iba1-siRNA injection, *p* = 0.999; sham vs. Iba1-siRNA injection, *p* = 0.999, Figure 4D).

In order to see if the reduced pain sensitivity was accompanied by a corresponding reduction in neuronal excitability, we performed whole-cell patch-clamp experiments on rat DRG neurons. Five days after SNL, compared to sham conditions (*n* = 12), the recorded neurons showed a significantly reduced resting membrane potential (mV) (*n* = 13, *p* = 0.050), and action potentials (AP) characterized by: (1) reduced total amplitude (mV) (*n* = 13, *p* = 0.022); (2) slower kinetics revealed by prolonged duration at the base (ms) (*n* = 13, *p* < 0.001) and during falling phase (ms) (*n* = 13, *p* < 0.001) and increased area at the base (mV*ms) (*n* = 13, *p* < 0.001), due, in particular, to increased area and rate of the falling phase (*n* = 13, *p* < 0.001 for both); and (3) significantly faster after-hyperpolarization (*n* = 13, *p* = 0.034 at 80% recovery) (Figure 4E–K and Supplemental Table S4 for additional parameters of AP and complete comparisons between conditions). Our findings are consistent with those of previous studies showing that damaged L5 DRG neurons have broader somatic APs with slower kinetics after L5 SNL [43,44,45]. Intriguingly, the treatment with Iba1-siRNA did not reduce any of the parameters of AP (Figure 4E–K and Supplemental Table S4), suggesting that the analgesic effect of local L5 delivery of Iba1-siRNA might be initiated locally, but it acts remotely. Previously, Djouhri and collaborators have shown that in vivo, L5 DRG neurons after SNL, axotomized, disconnected from their peripheral targets and degenerating, are less likely to make a significant contribution to peripheral pain pathogenesis, while intact L4 DRG neurons, which become hyperexcitable after SNL, possibly due to an overabundance of pro-inflammatory mediators and NGF diffused locally, are more responsible for the enhanced afferent input necessary for initiating and/or maintaining traumatic neuropathic pain [45]. Starting from this observation and from the fact that macrophages have an intense pro- and anti-nociceptive dialogue with the neurons [8,9], we continued to investigate if macrophages were switched to an anti-inflammatory, pro-analgesic phenotype after Iba1 silencing.

### 2.3. Iba1 Silencing Is Switching Macrophages from M1 Pro-Inflammatory Phenotype to M2 Anti-Inflammatory Phenotype

Since Iba1 is strongly up-regulated after peripheral nerve injury [24,25,26,27] and is associated with an alert, activated macrophages/microglia phenotype [46], we further investigated the consequences of Iba1 silencing on the expression of M1 and M2 markers, which are specific for classically activated, usually pro-inflammatory (M1) and alternatively activated, usually anti-inflammatory (M2) macrophages [47]. Macrophages have the unique ability to switch between an M1 or an M2 phenotype due to a rapid metabolic switch in arginine metabolism [48,49]. Recent studies have shown that 7 days after SNI, separated macrophages from the DRG have the M2 phenotype [27], but for SNL, this aspect has not yet been explored.

**Figure 5:**
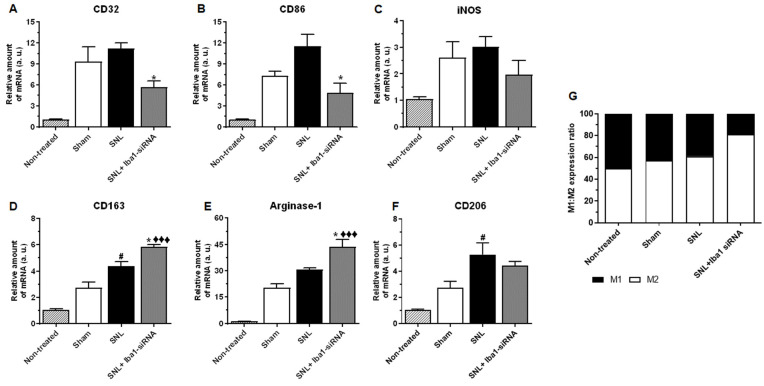
Iba1 silencing induces changes in the expression of genes associated with M1 and M2 macrophages. Bar graph representing qRT-PCR data for M1 markers (**A**–**C**) and for M2 markers (**D**–**F**). Of the M1 markers, CD32 and CD86 were significantly reduced by the Iba1-siRNA treatment (* *p* < 0.05), iNOS was not affected (*p* > 0.05) and all markers’ levels were not different compared to the sham condition (*p* > 0.05). Of the M2 markers, CD163 and Arginase-1 were significantly increased after Iba1-siRNA treatment compared to SNL (* *p* < 0.05) and sham conditions (^◆◆◆^ *p* < 0.001), while CD206 was not affected by Iba1 silencing (*p* > 0.05). # *p* represents comparisons between the SNL and sham conditions. (**G**) Macrophages with an M2 phenotype dominate after Iba1 silencing. When expressed as a ratio of M1/M2 cells, there is an obvious shift towards an M2 phenotype 5 days after SNL associated with intra-ganglionic delivery of Iba1-siRNA.

To test the expression of M1 (CD32, CD86 and iNOS) or M2 (CD163, Arginase-1 and CD206) markers [27], an RT-PCR test was run using a whole DRG tissue lysate (Figure 5; see also Supplemental Table S5 for numerical values of mRNA levels and for complete comparisons between conditions). The results showed that M1 markers CD32 and CD86 were significantly reduced after siRNA treatment compared to the SNL condition (~49% reduction, *n* = 3, *p* = 0.036 for CD32, Figure 5A, and ~58% reduction, *n* = 3, *p* = 0.010 for CD86, Figure 5B), but there was no significant reduction compared to the sham condition (Supplemental Table S5). For iNOS, however, there was no significant change compared to both SNL and sham conditions (Supplemental Table S5, Figure 5C).

On the other hand, M2 markers CD163 and Arginase-1 showed significantly increased levels after siRNA treatment compared to the SNL condition (~25% increase, *n* = 3, *p* = 0.023 for CD163, Figure 5D, and ~29% increase, *n* = 3, *p* = 0.022 for Arginase-1, Figure 5E). It further showed an even stronger increase when compared to the sham condition (~53% increase, *n* = 3, *p* < 0.001 for CD163, Figure 5D, and ~53% increase, *n* = 3, *p* < 0.001 for Arginase-1, Figure 5E). For CD206, however, there was no significant change compared to both SNL and sham conditions after Iba1 silencing (Supplemental Table S5, Figure 5F).

Because all macrophages express both M1 and M2 markers to different degrees, to see if the cells were skewed towards the M1 or M2 profile, we expressed the levels of M1 and M2 genes as the M1/M2 ratio. The results showed that most of the cells (82%) were skewed towards the M2 phenotype (Figure 5G).

**Figure 6:**
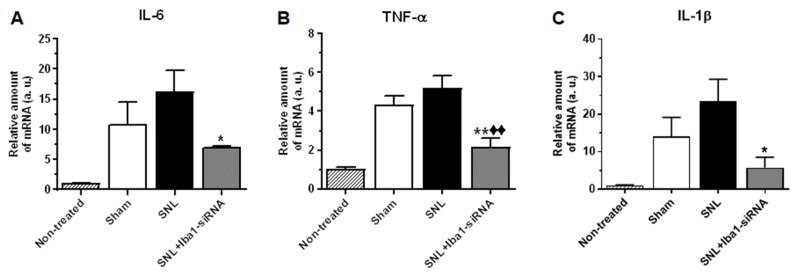
Iba1 silencing reduces the expression of pro-inflammatory mediators. Bar graph representing qRT-PCR data for pro-inflammatory cytokines IL-6 (**A**), TNF-α (**Β**) and IL-1β (**C**) significantly decreased after Iba1-siRNA treatment (* *p* < 0.05 for IL-6 and IL-1β and ** *p* < 0.01 for TNF-α). Only TNF-α decreased below sham levels after Iba1 silencing (^◆◆^ *p* < 0.01).

To further confirm the switch from an M1 to an M2 phenotype, we also investigated the influence of Iba1 silencing on the mRNA level of key pro-inflammatory cytokines IL-6, TNF-α and IL-1β, which were reportedly expressed in non-neuronal cells of the DRG [15,26,50] and have been previously implicated in neuropathic pain [6,15] (Figure 6; see also Supplemental Table S6 for numerical values of mRNA levels and for complete comparisons between conditions). The results confirmed our hypothesis and showed that all the pro-inflammatory cytokines were significantly reduced after siRNA treatment compared to the SNL condition (~56% reduction, *n* = 3, *p* = 0.026 for IL-6, Figure 6A; ~58% reduction, *n* = 3, *p* = 0.001 for TNF-α, Figure 6B; ~76% reduction, *n* = 3, *p* = 0.011 for IL-1β, Figure 6C). In contrast to other SNL studies, in which the evolution of pro-inflammatory mediators was evaluated at similar time points and where the SNL levels for IL-6, TNF-α and IL-1β were significantly higher than in the sham condition [51,52,53], in our study there were no significant differences between the two conditions (Supplemental Table S6). Opposite to the above-mentioned studies, in which the sham surgery involved nerve manipulation, in our study, the sham surgery involved nerve manipulation and random injections of 0.2× PBS or 400 nM scramble-siRNA into the L5 DRG, which subsequently induced a rapid macrophage response [8,38], also proven by increased mRNA and Iba1 protein levels in the sham condition compared to the non-treated condition (Figure 1). After Iba1 silencing, even though the pro-inflammatory mediators were lower than in the sham condition, only TNF-α showed a significant decrease (~49% reduction, *n* = 3, *p* = 0.008, Figure 6B and Supplemental Table S6).

**Figure 7:**
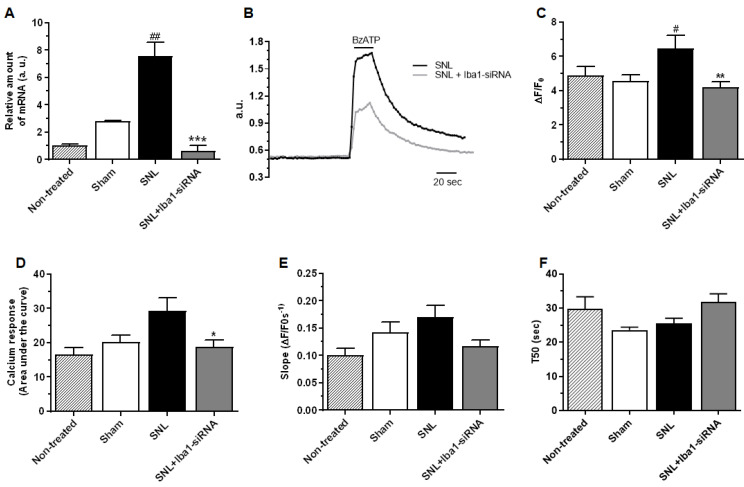
Iba1 silencing makes P2x7 receptors less active in SNL-activated macrophages. (**A**) Iba1 silencing significantly decreases caspase−1 in L5 DRG. Caspase−1 mRNA level was significantly up-regulated after SNL (^##^ *p* < 0.01 compared with the sham group) and was significantly down-regulated after injection of Iba1-siRNA (*** *p* < 0.001 compared with the SNL group). (**B**) Representative trace of [Ca^2+^]_i_ after activation of P2x7 receptors with 300 μM BzATP in macrophages isolated from SNL and SNL+ Iba1-siRNA L5 DRG. (**C**) Bar graph of mean ΔF/F0 induced via acute application of BzATP on +/− silenced L5 DRG macrophages, ** *p* < 0.01 compared with the SNL group, ^#^ *p* < 0.05 compared with the sham group. (**D**) Bar graph with mean area under the curve (AUC) as a measure of the [Ca^2+^]_i_ through P2x7 receptors after +/− Iba1 silencing, * *p* < 0.05. (**E**) Bar graph with mean decay slopes in +/− silenced L5 DRG macrophages, *p* > 0.05. (**F**) Bar graph of mean time to 50% decay in +/− L5 DRG macrophages, *p* > 0.05.

In order to understand what mechanism is activated inside macrophages to induce a decrease in pro-inflammatory mediators upon the Iba1 silencing-induced M1 to M2 switch, we explored whether this could be due to an altered/changed functioning of P2x7 receptors. Activation of P2x7Rs produces very fast changes of cytoskeleton in macrophages [54,55], which makes this receptor a highly probable target to be modulated by cytoskeleton reorganization upon Iba1 silencing. It was shown that in M1 polarized macrophages, the P2x7 receptor is involved in increased release of pro-inflammatory mediators via the NLRP3 inflammasome and caspase-1 cleavage, while in M2 macrophages, P2x7 activation was associated with a decreased release of pro-inflammatory mediators by uncoupling from the NLRP3 inflammasome [56,57,58]. To investigate whether the decrease in pro-inflammatory mediators upon the Iba1-silencing-induced M1 to M2 switch could be initiated by an Iba1-dependent mechanism of P2x7 uncoupling from inflammasome, we first measured the level of caspase-1 mRNA, which, if the P2x7 uncoupling from the inflammasome upon Iba1 silencing really occurred, should have been significantly reduced. The results showed that caspase-1 mRNA level significantly increased at the L5 DRG level after SNL (relative amount of caspase-1 mRNA in sham condition = 2.81 ± 0.03, *n* = 3, and after SNL = 7.57 ± 0.57, *n* = 3, ~63% increase, *p* = 0.001, Figure 7A), and that it was significantly reduced after Iba1 silencing (relative amount of caspase-1 mRNA in SNL + Iba1-siRNA = 0.63 ± 0.21, *n* = 3, ~92% decrease, *p* < 0.001, Figure 7A), to a lower level compared to the sham condition, very close to significance (*p* = 0.050).

Further on, we investigated the BzATP-activated current through P2x7 receptors in +/− Iba1-silenced DRG macrophages separated by positive CD11b immunopanning and cultured for 13–15 h to allow restoring cytoskeleton interactions with P2x7 receptors. P2x7 is expressed predominantly in cells of hematopoietic and immunologic origin, including microglia and macrophages [56], but it can also be expressed by satellite cells [59]. Nevertheless, since satellite cells do not express CD11b, they were not selected during the immunopanning procedure. The results showed the following: (1) a significant decrease in P2x7 current amplitude after Iba1 silencing (Figure 7B,C, mean ΔF/F0 in sham cells = 4.57 ± 0.36, *n* = 40, after SNL = 6.45 ± 0.75, *n* = 27, ~29% increase compared to sham, *p* = 0.027 and after SNL + Iba1-siRNA = 4.17 ± 0.33, *n* = 63, ~35% decrease compared to SNL, *p* = 0.002, to similar levels compared to sham condition (*p* = 0.999), accompanied by (2) a significantly reduced amount of calcium entering the cells after Iba1 silencing (Figure 7B,D, mean AUC in sham cells = 20.19 ± 1.96, *n* = 40, after SNL = 29.36 ± 3.81, *n* = 27, ~31% increase compared to sham, *p* = 0.054) and after SNL + Iba1-siRNA = 18.83 ± 1.88, *n* = 63, ~36% decrease compared to SNL, *p* = 0.010, to similar levels compared to sham condition (*p* = 0.999), and (3) no difference in recovery after Iba1 silencing (Figure 7B,E, mean slope (s) in sham cells = 0.14 ± 0.01, *n* = 40, after SNL = 0.16 ± 0.02, *n* = 27, *p* = 0.872 and after SNL + Iba1-siRNA = 0.11 ± 0.01, *n* = 63, *p* = 0.082) and for T50 (Figure 7B,F, mean T50 (s) in sham cells = 23.63 ± 0.80, *n* = 40, after SNL = 25.55 ± 1.48, *n* = 27, *p* = 0.999 and after SNL + Iba1-siRNA = 31.78 ± 2.44, *n* = 63, *p* = 0.180). These results contradict previous data showing that in vitro, P2x7 uncouples from the inflammasome in M2 macrophages without changes in its Ca^2+^ permeability or dye uptake [57,60], and suggest that in vivo, Iba1 silencing does more than uncoupling P2x7 from the inflammasome in M2 macrophages; it can also reduce the upstream P2x7 activation through a yet unknown mechanism.

**Figure 8:**
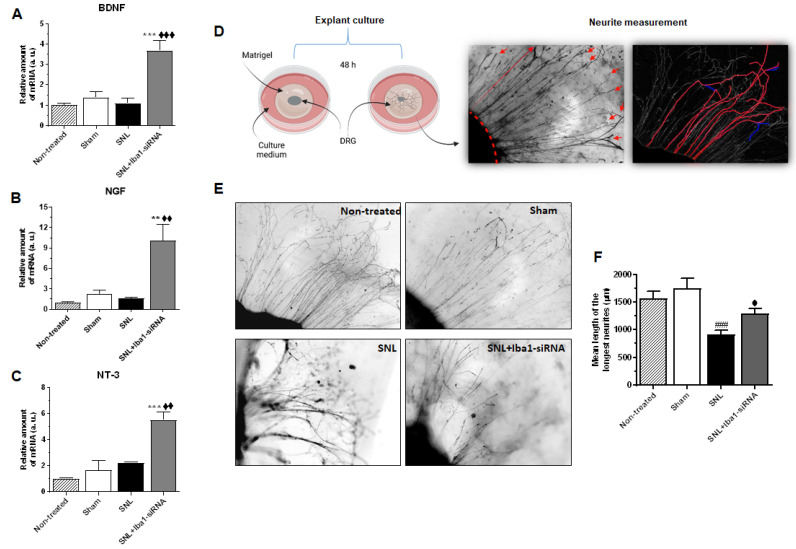
Iba1 silencing increases the expression of pro-regenerative mediators, which stimulate neurites’ regrowth. Bar graph representing qRT-PCR data for BDNF (**A**), NGF (**B**) and NT-3 (**C**) significantly increased after Iba1-siRNA treatment compared to SNL (*** *p* < 0.001 for BDNF and NT-3, ** *p* < 0.01 for NGF) and sham conditions (^◆◆◆^ *p* < 0.001 for BDNF, ^◆◆^ *p* < 0.01 for NGF and NT-3, and ^◆^ *p* represents comparisons between SNL+Iba1-siRNA and sham conditions). (**D**) Diagram showing the main steps in the neurite measurement assay: L5 DRG were cultured in Matrigel for 48 h, after which βIII-tubulin-stained processes were analyzed with NeuronJ (created with BioRender.com (accessed on 16 February 2023)). The dashed red line marks the edge of the DRG, and the red arrows point to the endings of individual neurites. (**E**) Representative inverted field images of adult L5 DRG explants after 48 h in culture. (**F**) Bar graph representing mean length of the longest neurites of L5 DRG neurons in different experimental conditions, indicating that their length was significantly reduced after SNL (^###^ *p* < 0.001), while after Iba1 silencing there is a 30% recovery, although not yet significant (*p* > 0.05).

Based on the observations that Iba1 silencing switches resident macrophages towards an M2 mode, we further hypothesized that since macrophages near axotomized neuronal cell bodies have a critical role in stimulating nerve regeneration [26,61], Iba1-silenced macrophages might also secrete factors that can accelerate nerve recovery and regeneration after SNL. Therefore, we investigated the influence of Iba1 silencing on the mRNA level of pro-regenerative factors BDNF, NGF and NT-3, which can be secreted by macrophages [62,63], were shown to contribute to sciatic nerve regeneration [64] and were associated with the pro-regenerative phenotype of macrophages in the conditioning injury model [26]. The results (Figure 8 and Supplemental Table S6 for numerical values of mRNA levels and for complete comparisons between conditions) showed that all the pro-regenerative factors significantly increased after Iba1 silencing compared to the SNL condition (~71% increase, *n* = 3, *p* < 0.001 for BDNF, Figure 8A; ~84% increase, *n* = 3, *p* = 0.001 for NGF, Figure 8B, and ~59% increase, *n* = 3, *p* = 0.001 for NT-3, Figure 8C and Supplemental Table S6). There was also a significant increase for all the pro-regenerative factors compared to the sham condition (~62% increase, *n* = 3, *p* < 0.001 for BDNF, Figure 8A; ~77% increase, *n* = 3, *p* = 0.001 for NGF, Figure 8B, and ~70% increase, *n* = 3, *p* < 0.001 for NT-3, Figure 8C and Supplemental Table S6).

To investigate whether the increased pro-regenerative factors do boost regeneration of DRG neurons, we performed a neurite assay on adult L5 DRG explants, cultured for 48 h, 5 days after Iba1-siRNA treatment (Figure 8D). The results have shown that SNL induced a significantly reduced length of neurites compared to the sham condition (mean neurites’ length (in μm) for the sham group = 1749 ± 185.2, *n* = 60, and after SNL = 911.9 ± 74.89, *n* = 53, ~48% reduction, *p* < 0.001, Figure 8E,F). Although the length recovered 30% after Iba1-siRNA treatment compared to the SNL group, it did not reach significance at this time point (mean neurites’ length (in μm) after siRNA treatment = 1294 ± 87.87, *n* = 60, *p* = 0.121, Figure 8E,F), and it was 26% below the sham values (*p* = 0.036, Figure 8E,F).

**Figure 9:**
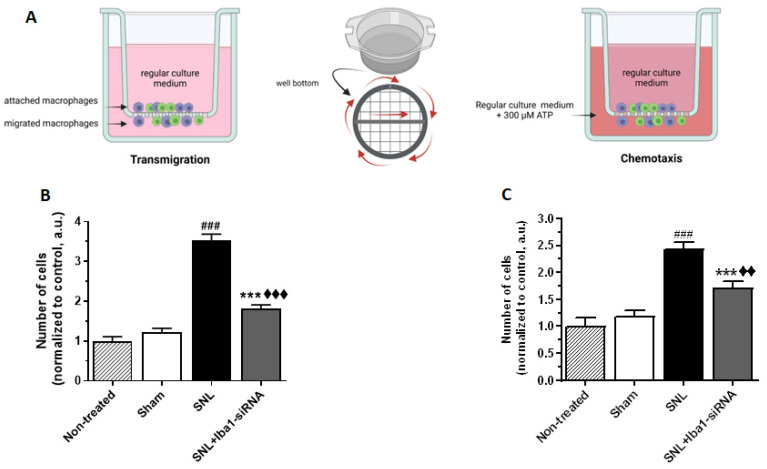
Iba1 silencing reduces the migration of rat resident macrophages. (**A**) Diagram showing the experimental setup for the transmigration and chemotaxis assays performed in Boyden chambers on L5 DRG macrophages (created with BioRender.com (accessed on 16 February 2023)). Red arrows near the chamber insert indicate the placement and direction of pictures taken for analysis. Bar graphs representing mean number of +/− silenced L5 DRG macrophages tested in different migration conditions: transmigration (free migration) (**B**) and chemotaxis (towards 300 µM ATP as chemotactic factor) (**C**). For both types of migration, the number of migrating macrophages significantly increased after SNL (^###^ *p* < 0.001) and significantly decreased after Iba1 silencing (*** *p* < 0.001), although it did not revert back to the sham levels (^◆◆◆^ *p* < 0.001 for transmigration and ^◆◆^ *p* < 0.01 for chemotaxis).

According to in vitro three-dimensional migration and in vivo tracking of fluorescently labeled macrophages performed on mice peritoneal macrophages or on human blood macrophages, it was shown that M1-activated macrophages have a strong substrate adhesion, which translates into weak 3D migration, while M2-activated macrophages, which have moderate substrate adhesion, are more motile [65,66]. To investigate if this was the case with the L5 DRG macrophages after Iba1 silencing, we performed an in vitro migration test on macrophages isolated by positive CD11b immunopanning from L5 DRG (Figure 9A). A double immunostaining with Iba1 and ED1 confirmed that most of the isolated cells were resident Iba1 (+) cells (Supplemental Figure S7).

The results have shown that, contrary to the data in the literature, SNL-activated/M1 macrophages migrated more both in free migration (transmigration) (mean number of migrating cells after sham surgery = 1.21 ± 0.10, *n* = 3, and after SNL = 3.52 ± 0.15, *n* = 3, ~65% increase, *p* < 0.001, Figure 9B) and in directed migration (chemotaxis) (mean number of migrating cells after sham surgery = 1.19 ± 0.10, *n* = 3, and after SNL = 2.43 ± 0.13, *n* = 3, ~51% increase, *p* < 0.001, Figure 9C). After siRNA treatment, the Iba1-silenced/M2 macrophages migrated significantly less both in transmigration (mean number of migrating cells = 1.82 ± 0.08, *n* = 3, ~48% decrease, *p* < 0.001, Figure 9B) and in chemotaxis (mean number of migrating cells = 1.70 ± 0.12, *n* = 3, ~30% decrease, *p* < 0.001, Figure 9C). However, the number of cells was still significantly higher compared to the sham condition (*p* < 0.001 for transmigration and *p* = 0.008 for chemotaxis). These results confirm our previous observation on BV2 microglia, namely that Iba1 silencing reduces migration [37], and also suggest that interfering with the cytoskeleton is another way of inducing the M2 phenotype in macrophages.

## 3. Discussion

Resident macrophages from DRG are critically involved in the development of nerve injury-induced hypersensitivity [8,15,24,25,26,27]. Here we show that intra-ganglionic delivery of Iba1-siRNA, administered at the time of the lesion, takes 5 days to induce an M1 to M2 switch in the DRG resident macrophages. Moreover, it reduces the secretion of pro-inflammatory cytokines and P2x7 currents, increases the secretion of pro-regenerative factors, which initiate the regrowth of adult DRG neurites, and reduces SNL-induced neuropathic pain.

Contrary to our hypothesis that Iba1-siRNA will prevent clustering of Iba1 (+) macrophages around neurons, the macrophages continued to cluster around all DRG neurons, with a preference for large NF200 (+) neurons. Most likely, this response is the expression of an increased sensitivity to traumatic injury of large DRG neurons [43,45,67] that attracted more macrophages around them by a CCL2- or CSF1-dependent chemotaxis [15,68]. On the other hand, the reduction of CGRP (+) and IB4 (+) neurons could have also contributed (i.e., 50–75% for CGRP (+) neurons and up to 87% for IB4 (+) neurons in L5 DRG, two weeks after SNL) [69,70]. In spite of this neuronal loss, SNL induced the clustering of Iba1 (+) macrophages around all types of DRG neurons 5 days after the lesion occurred, in contrast to SNI, where only large NF200 (+) neurons were surrounded by Iba1 (+) macrophages 7 days after the lesion occurred [25], suggesting that the clustering of macrophages around DRG neurons follows a lesion-specific pattern.

Interestingly, the ring-like clusters of macrophages were of different “strengths”, irrespective of the number of cells. Even though NF200 (+) neurons are more affected by the SNL injury and could have attracted more macrophages, in practice, there was no difference in the number of macrophages around them compared to CGRP (+) and IB4 (+) neurons. However, after Iba1 silencing, only the rings around NF200 (+) were “looser”, suggesting that spatial interactions between neurons and macrophages differ according to neuronal type. Such a different response did not have a different impact on pain sensitivity since the efficacy of reducing mechanical and cold allodynia was similar. In a previous study, it was shown that after SNI, macrophages that form rings around large neurons [25] contribute decisively to tactile allodynia [16]. In contrast, the same macrophages that form fewer rings around small C-fiber neurons [25] had no contribution to SNI-induced cold allodynia [16]. In our study, all neuronal types had rings of macrophages around them, and after Iba1 silencing, there was a reduction of both mechanical and cold allodynia, which suggests that for SNL, macrophages may contribute to both types of pain sensitivity. In addition, the altering of chemical communication between neurons and macrophages [8,9] seems to be critical for an analgesic effect rather than the loosening of the rings of macrophages around neurons.

It was previously shown that approximately 75% of DRG macrophages were in contact with neurons 7 days after sciatic nerve transection [26], and that most of the trigeminal neurons showed contact-like structures with Iba1 (+) cells infiltrated under a disrupted satellite glial cell sheath 5 to 7 days after infraorbital nerve ligation [71]. According to our electron microscopy study, 5 days after SNL, macrophages in the ring did not actually touch other macrophages, although gap junctions with satellite cells cannot be definitively excluded. Some of the macrophages infiltrated underneath the satellite cell sheath in an “*in-pocket*” position, as described in DRG 14 days after SNL [72] and in the trigeminal ganglia 7 days after infraorbital nerve ligation [71]. Intriguingly, we noticed that some macrophages were completely engulfed in the neuronal cytoplasm. DRG neurons from chick embryos (E11) and adult Wistar rats can phagocytose microspheres [73], and in rats, they have an increased uptake of fluorescein after SNL [74]. However, in our case, since no sign of lysis was detected inside the neurons, the engulfment process seemed more similar to emperipolesis [75] than to phagocytosis. The emperipolesis, shown before for lymphoid cells in vagal efferent neurons [76], but mostly related to cancer cells [77,78], was an unexpected finding in our study, and its physiological significance is unclear. We can speculate that macrophages inside the neurons could phagocytose injured organelles in order to allow the neuron to redirect its entire cellular activity towards healing, but this theory has no evidence in this study and will require additional data.

The most important observation of this study is that it was possible to induce, in vivo, an M1 to M2 switch in macrophages’ phenotype by interfering with Iba1, a cytoskeletal protein, with analgesic consequences. Even though the in vivo silencing efficacy was slightly reduced compared to our previous in vitro study on BV2 cells (Gheorghe et al., 2020 [37]) (i.e., mean silencing efficacy ~53% efficacy in vivo vs. ~65% in vitro), most likely due to the one nucleotide mismatch between the Iba1-siRNA and rat Iba1/Aif1 mRNA, our results reinforce previous studies that for local delivery, naked siRNA administered in vivo could be almost as efficient as polyethylenimine-complexed siRNA [79].

Switching macrophages to the M2 phenotype is a highly desirable approach for treating pain, and it was induced by IL-4 resulting in analgesic effects [80,81], with parthenolide resulting in repairing effects on spinal cord injury [82], or by altering the extracellular matrix structure [83]. For the first time, we show that this switch can be performed by interfering with the cytoskeleton via Iba1.

The M1 to M2 switch was additionally confirmed by a reduced release of pro-inflammatory cytokines, possibly associated with reduced P2x7 currents. P2x7Rs, associated with very fast changes of cytoskeleton as the cell is moving [54,55,84], is one of the most potent activators of the NLRP3 inflammasome, a key complex that initiates and boosts cytokine release via caspase-1 [56]. In M1 macrophages, the P2x7 receptor and NLRP3 coupling increase the release of pro-inflammatory mediators, while the opposite happens in M2 macrophages [57,58]. In our study, Iba1 silencing was associated with reduced P2x7 currents similar to our previous study in BV2 cells [37], which, combined with reduced caspase-1 levels, suggests that P2x7 impaired functioning may also contribute to the reduced release of pro-inflammatory cytokines.

In contrast to data showing that M2 macrophages are more motile than M1 macrophages [65,66], our Iba1-silencing-induced M2 macrophages migrated less. In vitro, M1 macrophages are less motile because they are tightly fixed to the substrate, with diffuse F-actin throughout the cytoplasm, while M2 macrophages are more mobile because they have fewer extensions and F-actin is tightly compacted close to the nucleus [57,85]. Previously, we have shown similar M2-type cytoskeleton reorganization in Iba1-silenced BV2 microglia [37], associated with reduced migration, which possibly occurred in Iba1 (+) macrophages as well. Nevertheless, these changes take time to develop since the Iba1 (+) rings around DRG neurons were still formed 5 days after SNL, despite Iba1 silencing at the time of the lesion.

Iba1-silencing-induced M1 to M2 switch did not reduce the SNL-induced hyperexcitability of L5 DRG neurons, contradicting another part of our hypothesis. L5 DRG neurons after SNL, with their peripheral connections severed, are less likely to make a significant contribution to peripheral pain pathogenesis [45], while the neighboring intact L4 neurons become hyperexcitable and painful, possibly due to increased inflammatory mediators [6], to the availability/transport of NGF to their soma and lowered pH [45], or due to pro-nociceptive miRNAs shed by macrophages [8]. In our study, Iba1 silencing reduced the secretion of pro-inflammatory mediators and thus possibly contributed to the Iba1 silencing-induced analgesia by a reduced activation of intact L4 DRG neurons. However, the contribution of L4 DRG neurons to Iba1 silencing-induced analgesia was beyond the purpose of this study because their role needs to be studied in vivo and not in vitro when the chemical environment is lost [45]. This Iba1 silencing-induced analgesia mechanism is more plausible than a possible reduction in L5 DRG neurons contribution to central sensitization [3], since the neurons’ excitability was not reduced and since BDNF, which acts as a secondary mediator between DRG neurons and the spinal cord, contributing to the establishment of a central sensitization process [86], was significantly increased after Iba1 silencing. It is not very clear at this stage how the Iba1 silencing was able to override the well-known pro-nociceptive effects of BDNF [87] and NGF [88], whose levels were increased after Iba1-siRNA treatment with beneficial effects on neurites’ regeneration but without nociceptive effects. We can speculate that the up-regulation of BDNF activated some anti-nociceptive mechanisms, since it was shown that over-expression of BDNF in the rat spinal cord was associated with amelioration of chronic neuropathic pain after partial nerve injury [89] and that a chronically applied, low local dose of BDNF supplied by transplanted cells near the spinal dorsal horn was able to reverse the development of chronic neuropathic pain following CCI [90]. A spinal release of GABA [91] or the activation of a clonidine-like noradrenergic pathway-induced analgesia [92] might explain the putative anti-nociceptive mechanism of BDNF in our experimental setup, but this requires further investigation. A potential limitation of our work is that we only used adult male rats to establish the animal model, so there may be a gender bias since recent research has found a sex difference in pain processing [93].

M2 macrophages at the DRG level were already described days after different peripheral nerve lesions (i.e., 7 days post SNI [27], 3 and 7 days post transection [94,95] and 3 days post partial sciatic nerve ligation [96]), indicating that macrophages may switch to the M2 phenotype after a lesion, regardless. Nevertheless, at the same time point, the pain hypersensitivity is quite high [97,98], suggesting that although macrophages may start to switch towards a healing, regenerative phenotype, they do that at the expense of increasing pain. In our study, after Iba1 silencing, the resident macrophages switched from the M1 to M2 phenotype 5 days after SNL. Furthermore, they do not leave the DRG but stay around neurons because of reduced migration and contribute to their healing and regeneration, as indicated by reduced pain and initiated neurite growth. Similar healing effects were suggested for M2 macrophages in the trigeminal ganglia 7 days after the infraorbital nerve lesion [71].

In conclusion, we show for the first time that it is possible to switch in vivo DRG macrophages from an M1 to an M2 phenotype using a naked, customized siRNA targeted against the Iba1 cytoskeleton protein, delivered inside the ganglia. If the silencing is initiated at the time of the lesion, in a few days, macrophages undergo physiological transformations (i.e., reduced secretion of pro-inflammatory mediators and increased secretion of pro-regenerative factors), which will accelerate the neuron-macrophage interactions required for reducing pain and neurite regrowth. Our results could be significant for other pathologies in which Iba1 (+) cells, either macrophages or microglia, are involved.

## 4. Materials and Methods

### 4.1. Animals and Experimental Design

All experimental procedures and protocols were carried out in accordance with Directive 2010/63/EU and Romanian Law 43/2014 on animal use for scientific purposes. The procedures and protocols were further conducted in accordance with the ARRIVE guidelines and were approved by the Ethics Committee of the Faculty of Biology, University of Bucharest, and the “Carol Davila” University of Medicine and Pharmacy (no 09/26.04.2021).

A total of 167 male Sprague Dawley rats (150–200 g, animal facility of “Ion Cantacuzino” National Institute, Bucharest, Romania) were randomly assigned to one of the following groups: (1) non-treated—no interventions; (2) sham—nerve exposed and manipulated + random injections of 0.2× PBS or 400 nM scramble-siRNA into the L5 DRG. Since in preliminary studies we noticed that for both Iba1 mRNA and protein expression there was no significant difference between sham-PBS and sham-scramble conditions, we decided to merge the animals in one sham group and considered it as our reference condition = control in all the experiments; (3) SNL—test group with L5 spinal nerve ligated and transected and (4) SNL+Iba1- siRNA test group injected with 400 nM Iba1-siRNA into the L5 DRG before SNL surgery. A graphical representation of the experimental design is presented in Supplemental Figure S1. To reduce the number of animals used for experiments, when possible, contralateral DRG from sham-operated animals were used for the non-treated group. The animals were housed individually in cages with ad libitum access to food and water in a climate-controlled room at a room temperature of 23 °C ± 0.5 °C and a 12 h light/dark cycle. The surgery was performed by the same experimenter. Three rats were excluded from the study: two died prematurely, post-operative deaths (groups 3 and 4), and one rat died due to ascites (group 4).

The same two customized siRNA molecules (Axolabs, Kulmbach, Germany) as previously published [37] were used in this study: Iba1-siRNA (sense: 5′-uGGAAAuGGAGAuAuCGAudTsdT-3′, antisense: 5′-AUCGAuAUCUCcAUUUCcAdTsdT-3′) and scramble-siRNA (sense: 5′-cuuAcGcuGAGuAcuucGAdTsdT-3′ and antisense: 5′-UCGAAGuACUcAGCGuAAGdTsdT-3′), with the following associated chemistry: 2′-O-methyl modifications are indicated with lowercase letters; phosphorothioate modifications are indicated with s and dT, indicating a DNA T base.

The Iba1-siRNA was designed based on the mouse mRNA molecule (NCBI Reference Sequence: NM_019467.3, https://www.ncbi.nlm.nih.gov/nuccore/NM_019467.3 (accessed on 1 October 2008)) and identified as compatible with rat allograft inflammatory factor 1 (Aif1) mRNA with the aid of the BLAST alignment tool. According to MUSCLE (Multiple Sequence Comparison by Log-Expectation), the antisense strand of Iba1-siRNA would hit the rat Aif1 mRNA sequence at 176 nucleotides from the initiation codon, with one nucleotide mismatch (Supplemental Figure S2). The open reading frame (ORF) analysis was performed using the NCBI nucleotide database in GenBank format (https://www.ncbi.nlm.nih.gov/nucleotide/ (accessed on 28 April 2021)). Since Aif1 is another name for the Iba1 protein, the siRNA will be referred to as Iba1-siRNA. Both siRNA molecules were formulated in 0.2× PBS and injected naked at the level of the ipsilateral L5 DRG, according to previous data on naked siRNA in vivo delivery to macrophages [79] and following the procedure described below. Previously, we established through dose–response studies that 40 nM Iba1-siRNA was the most effective in silencing Iba1 protein in BV2 microglia [37]. In this study, to overcome a possible degradation of siRNA in vivo, we used a 400 nM solution.

### 4.2. SNL Surgery and Intra-Ganglionic Delivery Procedure

Rats were anesthetized with 4% isoflurane (Rompharm, Otopeni, Romania) in air for induction of the anesthesia, and then maintained with 2% isoflurane. For SNL surgery, we followed a slightly modified version of the method previously described [41]. Briefly, the skin was incised, the right paravertebral region was exposed, and the connective tissue and muscles were removed to gain access to bone structures and the L5 intervertebral foramen. Next, the L6 transverse process was removed to expose the L5 and L4 spinal nerves, and the L5 nerve was ligated with a 6-0 silk suture and transected distally. All surgical procedures were performed on deeply anesthetized rats, and no surgery was performed on the left side.

For intra-ganglionic injection, the marginal laminar rim caudal to the L5 ganglion was removed using a small Rongeur to expose the caudal part of the L5 ganglion. Next, 0.2× PBS, scramble-siRNA or Iba1-siRNA were injected in a 2 μL bolus via a 29-gauge needle with a slightly bent tip [99], according to the experimental condition. The injection volume was the same as previously published [100].

On day 5 after surgery, animals were subjected to behavioral tests, then sacrificed, and the ipsilateral L5 DRG were collected for the tests described below. We chose day 5 because, according to our studies [24], on day 5, Iba1 mRNA was significantly increased in both DRG and spinal cord, with the former showing a greater uptick than the latter.

### 4.3. Behavioral Tests

Behavioral testing was performed one day before the surgery (to establish the baseline) and twice postoperatively, on the 3rd and 5th days. When moved to a new environment, the animals were left for 30 min undisturbed to habituate to the testing environment. Each testing day, mechanical and thermal hyperalgesia were evaluated by applying behavioral tests in the same sequence: dynamic plantar aesthesiometer, acetone and hot plate tests. Rats were placed on a mesh-wire surface in individual clear plastic enclosures, and stimuli were applied to the plantar skin of the right hind paw. A positive response was recorded if shaking, flinching, licking or withdrawing the paw occurred. Testing was carried out before surgery and post-injury by the same experimenter, who was blind to the treatment procedure. However, due to the deformity of the limb after SNL surgery, data could not be collected completely in a blind way. A facial expression scale, the rat grimace scale, with four facial action units [101], was used to confirm the animal’s welfare. N represents the number of animals used in each experimental group.

#### 4.3.1. Mechanical Stimulation

Test with dynamic plantar aesthesiometer (DPA). The mechanical stimulus was delivered to the plantar surface of the hind paw from below the floor of the testing chamber with a DPA (37450; Ugo Basile, Gemonio, Italy) that automatically stops when the animal withdraws the paw [102]. An increasing force using a rigid plastic filament (0.5 mm diameter) was applied in the middle of the plantar surface of the right hind paw. The force applied was initially below the detection threshold, increased from 1 to 40 g in 4 g/s steps over 10 s, and held at 40 g for an additional 10 s. The force applied to elicit a withdrawal response of the hind paw was defined as the mean of three measurements at 2 min intervals. The parameters recorded were the threshold and latency of withdrawal responses. In all experimental conditions, data were normalized to the baseline of each animal and to the mean value of the non-treated condition.

#### 4.3.2. Thermal Stimulation: Acetone Test

Acetone (32201; Honeywell, Charlotte, NC, USA) was expelled from a syringe attached to tubing to form a drop that was applied to the mid-plantar surface of the right hind paw with no contact between the tubing and the skin, as previously described [40]. The solution was applied 3 times spaced at least 2 min apart, and the number of positive responses within 60 s of acetone applications was recorded and averaged [103]. A positive response was recorded if shaking, flinching, licking or withdrawal of the paw were present. In all experimental conditions, data were normalized to the baseline of each group and to the mean value of the non-treated condition.

#### 4.3.3. Thermal Stimulation: Hot Plate Test

The animals were placed on a metal hot plate (35100; Ugo Basile, Gemonio, Italy) at 48 °C for an evaluation period of 30 s [104]. The total reaction time was measured from when the rat was placed on the plate until the first response was displayed as licking, lifting the paw or jumping on the plate. Lifting for normal locomotion was excluded. Animals were tested 3 times spaced at least 10 min apart. In all experimental conditions, data were normalized to the baseline of each animal and to the mean value of the non-treated condition.

### 4.4. Quantitative Reverse Transcription—Polymerase Chain Reaction (qRT-PCR)

DRG freshly collected 5 days after surgery (3 × L5 DRG/sample) were placed in RNAlater solution (R0901; Sigma, St. Louis, MO, USA) before proceeding to RNA isolation. N represents the number of animals used in each experimental group.

For confirming Iba1 silencing, total RNA was extracted using the GenEluteTM Mammalian Total RNA Miniprep Kit (RTN70; Sigma, St. Louis, MO, USA) and on-column DNase I digestion set (DNASE70; Sigma, St. Louis, MO, USA) according to the manufacturer’s instructions. RNA concentrations and purity were determined using spectrophotometric measurements at 260 and 280 nm using a Beckman Coulter DU730 (Beckman Coulter, Brea, CA, USA) spectrophotometer. The mean value of the A260/A280 ratio was 2 ± 0.04 (*n* = 15; for this test only, *n* represents the number of samples; in each sample, 3 DRG per experimental group were pooled from 3 different animals), in range with the recommended values for the isolation kit. The integrity of the RNA samples was confirmed on an Agilent 2100 Bioanalyzer (Agilent, Santa Clara, CA, USA); only samples with an RNA integrity number ≥ 8 were processed further. Reverse transcription was performed using the High-Capacity cDNA Reverse Transcription Kit (4368814; Life Technologies, Carlsbad, CA, USA) and an RNase inhibitor solution (N8080119; Life Technologies, Carlsbad, CA, USA). The relative abundance of Iba1 mRNA was assessed by qRT-PCR using TaqMan methodology on an ABI Prism 7300 Sequence Detection System (Applied Biosystems, Waltham, MA, USA). Amplification reactions were carried out for 50 cycles in triplicate using specific primers for Iba1 (Mm00479862_g1: Thermo Scientific, Waltham, MA, USA). Quantitative RT-PCR data were evaluated using the 2(-Delta Delta C(T)) method [105] and were normalized to the internal control and to the mean value of the non-treated condition.

To confirm the expression of M1 and M2 markers, pro-inflammatory cytokines, pro-regenerative mediators and caspase-1, the protocol was similar to the one above, except for the following: RNA isolation was performed using the RNeasy Micro kit (74004; Qiagen, Hilden, Germany), and qRT-PCR was performed on a ViiA™ 7 Real-Time PCR System ViiA7 system (Applied Biosystems, Waltham, MA, USA) using SYBR Select Master Mix (4472908; Thermo Scientific, Waltham, MA, USA). The primers’ sequences for M1 markers (CD32, iNOS, CD86), M2 markers (CD163, CD206, Arginase-1), pro-inflammatory cytokines (IL-6, TNFα and IL-1β), pro-regenerative factors (BDNF, NGF and Neurotrophin-3), caspase-1 and the reference genes used in this study are presented in Supplemental Table S1. The data were normalized to the internal controls 18S (4333760F; Life Technologies, Carlsbad, CA, USA) and YWHAZ (Rn00755072_m1; Thermo Scientific, Waltham, MA, USA).

### 4.5. Western Blotting

DRG freshly collected 5 days after surgery were subjected to lysis with RIPA buffer containing 25 mM Tris-HCl pH 7.6, 150 mM NaCl, 1% NP-40, 1% sodium deoxycholate, 0.1% SDS (89901; Thermo Scientific, Waltham, MA, USA) and 1× protease inhibitor (11836170001; Roche, Basel, Switzerland). Further on, proteins were mechanically extracted from single L5 DRG with a mixer mill MM 400 (Retsch, Verder Scientific, Haan, Germany) for 1 min at a frequency of 30 Hz. The lysates were cleared by centrifugation at 10,000× *g* at 4 °C, and the supernatant was stored at −80 °C until further processing.

The total amount of protein was determined using the BCA assay (23225; Life Technologies, Carlsbad, CA, USA), and equal amounts of extracted proteins were separated on 15% SDS-PAGE and transferred to a PVDF membrane. The membranes were probed with rabbit anti-Iba1 (1:1000, ab178846; Abcam, Cambridge, UK) and mouse anti-GAPDH (1:1000, sc-32233; Santa-Cruz Biotechnology, Dallas, TX, USA) antibodies overnight at 4 °C, washed and incubated with secondary antibodies coupled with HRP: anti-rabbit (1:10,000, sc-2357; Santa-Cruz Biotechnology, Dallas, TX, USA) and anti-mouse (1:10,000, sc-516102; Santa-Cruz Biotechnology, Dallas, TX, USA). Visualization was made with the ECL Prime Western Blotting System (RPN2232; GE Healthcare, Chicago, IL, USA) and Pierce ECL Western blotting substrate (32106; Thermo Scientific, Waltham, MA, USA). If not otherwise mentioned, all the chemicals and reagents were from Santa Cruz Biotechnology. Band intensity was quantified using Image J 1.53e (Wayne Rasband, National Institutes of Health, Bethesda, MD, USA) software, and the results were normalized to the mean value of the non-treated condition. N represents the number of animals used in each experimental group.

### 4.6. Electron Microscopy (EM)

DRG freshly collected 5 days after surgery were processed according to the procedure briefly described in Supplemental Table S2. The tissue samples were cut into thin sections (φ = 1 μm) using a Leica EM UC7 (Leica Microsystems, Wetzlar, Germany) ultra-microtome, stained with 1% toluidine blue and used for histologic examination and orientation procedures [106]. After the orientation procedure, the tissue samples were cut into ultrathin sections (φ = 60–80 nm), which were harvested on 3 mm copper grids and stained with uranyl acetate 1% and Reynolds solution. The images were acquired using a FEI Morgagni 268 Transmission Electron Microscope (FEI Company, Hillsboro, OR, USA) at 80 kV with a MegaView III (Olympus, Hamburg, Germany) CCD camera. For the EM analysis, we used 3 rats for each experimental group.

### 4.7. Immunohistochemistry (IHC)

Five days after the surgery, the animals were anesthetized with 5% isoflurane in air and perfused through the ascending aorta via the left ventricle with 1× PBS followed by 4% paraformaldehyde (PFA). The L5 ganglion was removed and post-fixed overnight in 4% PFA, then washed with 1× PBS, followed by incubation with 30% sucrose (48–72 h) for cryoprotection. Samples were embedded in optimal-cutting-temperature freezing medium (05-9801; Bio-Optica, Milan, Italy) and sectioned on a cryostat in 10 μm slices, which were placed on Superfrost Ultra Plus^®^ glass slides (J3800AMNZ; Thermo Fisher Scientific, Waltham, MA, USA).

Sections were washed with 1× PBS, blocked in 4% normal goat serum and then stained overnight with the antibody against Iba1 (1:1000, rabbit, 019-19741; Wako Chemicals, Richmond, VA, USA), ED1 (1:200, mouse, ab31630; Abcam, Cambridge, UK), MHC-II (1:200, mouse, ab6403; Abcam, Cambridge, UK), NF200 (1:1000, mouse, N0142; Sigma, St. Louis, MO, USA), biotinylated isolectin B4 (1:100, B-1205, Vector Laboratories, Newark, CA, USA), CGRP (1:400, mouse, ab81887; Abcam, Cambridge, UK) or β III tubulin (1:1000, mouse monoclonal, ab78078, Abcam, Cambridge, UK). For the IB4 and β III tubulin stainings, a specific heat treatment for antigen retrieval was applied: before proceeding to the blocking step, DRG sections were microwaved for 1 min and 30 s (≈800 W) in a preheated buffer (70 °C, 10 mM Tris Base, 1 mM EDTA solution, 0.05% Tween 20, pH 9.0).

Next, after washing thoroughly with 0.3% Triton X-100 in PBS, sections were incubated for 2 h with secondary antibodies (goat anti-rabbit Alexa Fluor 488 (1:1500, A-11008; ThermoFisher Scientific, Waltham, MA, USA), goat anti-mouse Alexa Fluor 568 (1:1500, A-11004; ThermoFisher Scientific, Waltham, MA, USA) or Streptavidin-Alexa Fluor 350 Conjugate (1:400, S11249, Invitrogen, Carlsbad, CA, USA). The sections were washed with 0.3% Triton X-100 in PBS and then 1× PBS before being cover-slipped using ProLong^TM^ Gold Antifade Mountant with or without DAPI (P36935, P36930; ThermoFisher Scientific, Waltham, MA, USA) and left to cure for 24 h at room temperature (RT) in the dark. Slides were examined using an Olympus IX73 fluorescence microscope equipped with a Hamamatsu ORCA-03G camera and processed with CellSens Dimension 1.11 software (Olympus Corporation, Tokyo, Japan). For the immunohistochemistry analysis, we used 8 sections/animal and 3 areas/section in *n* = 3 rats for each experimental group.

Cells were counted as having macrophage ring-like clusters when the perimeter of the neuron was at least 50% surrounded by macrophages, which were disposed closely to the neuronal surface. To quantify the ring area of Iba1 (+) macrophages around NF200 (+), CGRP (+), and IB4 (+) DRG neurons, we used a plugin written for ImageJ 1.37v (NIH, Bethesda, MD, USA) (Supplemental Figure S3). Mean fluorescence was considered an indicator of the macrophage density around the neurons.

### 4.8. Immunopanning and Cultures of Macrophages

To obtain purified macrophage cultures from DRG, one day prior to collecting the cells, 13 mm glass coverslips were treated with 6 μg/mL of goat anti-mouse IgG secondary antibody (ab6708; Abcam, Cambridge, UK) in 50 mM Tris buffer (pH 9.5) for 2 h at 37 °C. Then, following 1× PBS washes, coverslips were incubated overnight at RT with 1 μg/mL of mouse anti-rat CD11b antibody (MCA275G; Biorad, Hercules, CA, USA) and 2 mg/mL of peptone from milk solids (P6838; Sigma, St. Louis, MO, USA) in 1× PBS.

L5 DRG were removed and dissociated with 1.5 mg/mL collagenase IA (C9891; Sigma, St. Louis, MO, USA) and 2.5 mg/mL dispase (1710541; Gibco, Waltham, MA, USA) for 1 h at 37 °C. The cell suspension was plated on the coverslips pre-coated with antibodies for 20 min at RT in Dulbecco’s Modified Eagle’s Medium (10-013-CVR; Corning, NY, USA) supplemented with 10% fetal bovine serum (102701060; Life Technologies, Carlsbad, CA, USA) and 1% Penicillin/Streptomycin (P4458; Sigma, St. Louis, MO, USA). Once the incubation was completed, coverslips were washed with 1× PBS to remove unbound floating cells and then macrophages were cultivated for 13–15 h.

### 4.9. Migration Transwell Assay

The ability to migrate after Iba1 silencing was evaluated using the 24-well Transwell assay with 8 μm GreinerBio-One ThinCert^TM^ Inserts (662638; Greiner Bio-One, Frickenhausen, Germany). Macrophages selected by immunopanning with CD11b antibodies were cultivated at 2 × 10^4^ cells in the upper chamber of the transwell in 200 μL of medium. A volume of 600 μL medium was added in the lower chamber, and then the cells were incubated for 24 h.

To test transmigration, normal culture medium was added both in the transwell and the lower chamber, while for chemotaxis, a conditioned medium containing 300 μM ATP was added in the lower chamber. After 24 h, the inserts were fixed with 4% PFA for 5 min, washed with 1× PBS and stained against Iba1 (1:1000, rabbit, 019-19741; Wako Chemicals, Richmond, VA, USA) and with 2 μg/mL Hoechst. The round bottom inserts were photographed all around and on a horizontal plan, starting from the middle of the insert and then from one end to the other. Experiments were carried out in triplicate, and the results were normalized to the non-treated cells. To quantify the number of Iba1 (+) macrophages that migrated through the 8 μm pores, we used an in-house plugin written for ImageJ 1.37v (NIH, USA) that selected Iba1 (+) macrophages from images with cells co-stained against Hoechst and Iba1. N represents the number of animals used in each experimental group.

### 4.10. Electrophysiological Recordings

Whole-cell current-clamp recordings were acquired from DRG neurons originating from L5 dorsal root ganglia and cultured for 16–18 h on glass coverslips treated with poly-D-lysine (P0899, Sigma, St. Louis, MO, USA) as previously described [45]. The electrodes were obtained from borosilicate glass capillaries (GC150T, Harvard Apparatus, Cambridge, MA, USA), pulled with a vertical micropipette puller (Pull-100, World Precision Instruments, Sarasota, FL, USA) and heat polished to obtain a resistance between 2 and 4 MΩ. After the formation of a tight seal (>1 GΩ), membrane resistance, series resistance and capacitance (Cm) were determined; the recordings were conducted only when the access resistance was lower than 10 MΩ.

The extracellular solution contained (in mM): NaCl 140, KCl 4, MgCl_2_ 1, CaCl_2_ 2, HEPES 10, NaOH 4.54 (pH 7.4 with NaOH at 25 °C), to which glucose (7.4 mM) was added on the day of the experiment. The 0.2 μm filtered intracellular solution containing (in mM): NaCl 5, KCl 130, MgCl_2_ 2, CaCl_2_ 1, HEPES 10, EGTA 10 (pH adjusted to 7.3 with KOH) was used to fill the electrodes. Cells were visualized with a Nikon Eclipse TE300 inverted microscope (Nikon, Tokyo, Japan). The recordings were made at 37 °C (maintained with a TC202A (Harvard Instruments, Holliston, MA, USA) temperature controller), using a resistor-feedback WPC-100 patch-clamp amplifier (ESF Electronic, Goettingen, Germany), and were filtered at 3 kHz and digitized at 10 kHz with the DigiData 1322A A/D interface (Molecular Devices, San Jose, CA, USA). Action potentials (AP) were evoked with a 5 ms current injection step protocol from 100 to 500 pA and recorded with pClamp 8 software (Molecular Devices, San Jose, CA, USA). Action potential parameters (as described in Supplemental Figure S4) were extracted using in-house-made Python scripts. The .abf files were directly imported into Python using thepyABF library [107]. N represents the number of cells analyzed from 3 animals in each experimental group.

### 4.11. Ratiometric Intracellular Ca^2+^ Imaging

To evaluate P2x7R functioning, macrophages from all conditions isolated by immunopanning were seeded on 13 mm coverslips and maintained for an additional 13–15 h until recording. The rationale for this incubation time was that, in order to properly activate P2x7 receptors, the macrophages needed to be attached to the substrate with the cytoskeleton exposed and connected to P2x7 receptors.

On the day of the experiment, the cells were incubated for 30 min at 37 °C in an extracellular solution (see below) containing 2 mM Fura-2 AM and 0.02% Pluronic F-127 (F1221 and P6867; Life Technologies), mounted into a Teflon chamber (RC-40HP; Harvard Apparatus, USA) and visualized with a Nikon Eclipse TE300 inverted microscope (Nikon, Tokyo, Japan). The cells were illuminated at 340/380 nm with an Optoscan monocromator (Cairn Instruments, Faversham, UK), and the fluorescence changes were captured with a 12-bit CCD SensiCam camera (PCO, Kelheim, Germany). The data were recorded using Axon Imaging Workbench 4.0 (Indec Biosystems, Santa Clara, CA, USA) and analyzed with an Excel macro written in VSB. After background subtraction, the data were quantified as ΔF/F0, the area under the curve (AUC), the decay slope and the time of 50% decay (t50) (Supplemental Figure S5). N represents the number of cells analyzed from 3 animals in each experimental group.

The protocol consisted of one brief 20 s application of 300 µM BzATP (2′(3′)-O-(4-Benzoylbenzoyl) adenosine 5′-triphosphate), a specific agonist for P2x7 receptors [108], followed by 180 s washout with an extracellular solution that contained (in mM): NaCl 140, KCl 4, CaCl_2_ 2, MgCl_2_ 1, D-glucose 7.4 and 4-(2-hydroxyethyl)-1-piperazineethanesulfonic acid (HEPES) 10, adjusted with NaOH at pH 7.4.

### 4.12. Neurite Measurement Assay on DRG Explants

To assess the outgrowth of sensory neurons in response to injury, we evaluated neurite outgrowth in explanted ganglia from rats with or without injury. In order to increase adherence to the substrate and facilitate neurite growth, the glass coverslips were first treated with H_2_SO_4_ for 24 h, washed with distilled water and ethanol 100%, heat-dried over flame and kept in ethanol 100% until use. Five days after each experimental procedure, L5 DRG were removed, desheathed, placed on coverslips, and overlaid with 10 μL of Matrigel^®^ Basement Membrane Matrix (356234; Corning, NY, USA). Culture plates were placed in a 37 °C incubator for 10 min to allow gelling of the Matrigel before adding 500 μL of culture medium (1:1 mixture of 7.4 mM glucose DMEM and Hams’s F10 medium with 10% horse serum, 0.5% Penicillin/Streptomycin and 1% l-glutamine). After 48 h, explants were fixed and labeled with an antibody against β III tubulin (1:1000, mouse monoclonal, ab78078, Abcam, Cambridge, UK), followed by a 1 h incubation with goat anti-mouse Alexa Fluor 568 (1:1500, A-11004; ThermoFisher Scientific, Waltham, MA, USA) or goat anti-mouse Alexa Fluor 488 (1:1500, A-11001; ThermoFisher Scientific, Waltham, MA, USA). Next, coverslips were placed onto slides with Prolong Gold antifade (P36930; LifeTechnologies, Carlsbad, CA, USA), and the outgrowth was photographed using an inverted Olympus IX73 fluorescence microscope at 10× magnification and processed with CellSens Dimension 1.11 and Image J software. Neurite outgrowth was assessed using NeuronJ, an ImageJ plugin for neurite tracing and analysis [109], by measuring the processes taking place between the edge of the ganglion and the leading tip. For each DRG, the longest 20 processes were analyzed. Experiments were carried out in triplicate, and *n* represents the number of neurites analyzed in each experimental group.

### 4.13. Statistical Analysis

All data were given as means ± SEM. When three or more groups were compared, statistical significance was tested using one-way ANOVA with a Bonferonni post hoc test or a repeated measures ANOVA with Bonferonni post hoc test when the evolution in time of a parameter was compared between multiple groups. Two-tailed Student’s t-tests were used when comparing only two groups (GraphPad Prism 9.0 software). Different symbols were used according to the comparison: #—SNL vs. sham, *—SNL vs. SNL+Iba1-siRNA and ♦—SNL+Iba1-siRNA vs. sham. A value of *p* < 0.05 was considered to be statistically significant, with * *p* < 0.05, ** *p* < 0.01 and *** *p* < 0.001.

## Data Availability

The data that support the findings of this study are available upon reasonable request to the corresponding author.

## Supplementary Materials

The following supporting information can be downloaded at https://www.mdpi.com/article/10.3390/ijms242115831/s1.

## Abbreviations

ADP100	Duration of the action potential base
ADP_os_	Duration at the overshoot level
AHP	Action potential hyperpolarization amplitude
AHP_sub_	Subtracted voltage value from resting potential to AHP
Aif1	Allograft inflammatory factor 1
ANOVA	Analysis of variance
AP	Action potential
AP Amp	Action potential total amplitude
AP FP	Duration of the AP falling phase
AP OS	Action potential overshoot value
AP RPh	Duration of the AP rising phase
AP Th	Threshold potential
AUC	Area under the curve
BCA assay	Bicinchoninic acid assay
BDNF	Brain derived neurotrophic factor
BLAST	Basic Local Alignment Search Tool
BzATP	(2′(3′)-O-(4-Benzoylbenzoyl)adenosine-5′-triphosphate)
CaCl	Calcium Chloride
CCI	Chronic Constriction Injury
CCL2	Chemokine (C-C motif) ligand 2
CD163	Cluster of Differentiation 163
CD206	Cluster of Differentiation 206
CD32	Cluster of Differentiation 32
CD86	Cluster of Differentiation 86
CD 68 (ED1)	Cluster of Differentiation 68
Cd11b	Cluster of Differentiation 11b
CGRP	Calcitonin gene-related peptide
CSF1	Colony stimulating factor 1
DAPI	4′,6-diamidino-2-phenylindole
DNA	Deoxyribonucleic acid
DPA	Dynamic plantar aesthesiometer
DRG	Dorsal root ganglia
EGTA	1,2-bis(o-aminophenoxy)ethane-N,N,N′,N′-tetraacetic acid
E11	Embryonic day 11
EM	Electron microscopy
F-actin	Filamentous actin
FPh_area_	Area at the falling phase of action potential
GAPDH	Glyceraldehyde 3-phosphate dehydrogenase
HEPES	4-(2-hydroxyethyl)-1-piperazineethanesulfonic acid
HRP	Horseradish peroxidase
IB4	Isolectin B4
Iba1	Ionized binding adapter protein 1
ICC	Immunocytochemistry
IgG	Immunoglobulin G
IHC	Immunohistochemistry
IL-4	Interleukin 4
IL-1β	Interleukin 1β
IL-6	Interleukin 6
iNOS	inducible Nitric oxide synthase
KCl	Potassium Chloride
KOH	Potassium Hydroxide
LPS	Lipopolysaccharide
MAC1	Macrophage-1 antigen
MAFIA	Macrophage Fas-induced apoptosis
MgCl	Magnesium Chloride
MHC-II	Major histocompatibility complex class II
mRNA	messenger Ribonucleic acid
MUSCLE	MUltiple Sequence Comparison by Log-Expectation
NaCl	Sodium Chloride
NaOH	Sodium hydroxide
NCBI	National Center for Biotechnology Information
NF200	Neurofilament 200 kDa
NGF	Nerve growth factor
NLRP3	NOD-, LRR- and pyrin domain-containing protein 3 = Nucleotide-Binding Oligomerization Domain, Leucine Rich Repeat and Pyrin Domain Containing protein 3
NO	Nitric oxide
NT-3	Neurotrophin 3
ORF	Open reading frame
P2x7R	P2x7 receptor
PBS	Phosphate buffer saline
PFA	Paraformaldehyde
PVDF	Polyvinylidene difluoride
qRT-PCR	Quantitative Reverse Transcription–Polymerase Chain Reaction
RIPA	Radioimmunoprecipitation assay buffer
RNA	Ribonucleic acid
RP	Resting potential
RPh_area_	Area at the rising phase of action potential
S100A9	S100 calcium-binding protein A9
SDS-PAGE	Sodium dodecyl sulphate–polyacrylamide gel electrophoresis
SEM	Standard error of the mean
siRNA	Small interfering RNA
SNI	Spared nerve injury
SNL	Spinal nerve ligation
TNF-α	Tumor necrosis factor-α
VSB	Visual Basic for Applications
