# Supplementary material for: Switching Rat Resident Macrophages from M1 to M2 Phenotype by Iba1 Silencing Has Analgesic Effects in SNL-Induced Neuropathic Pain
Source: Int J Mol Sci. 2023 Oct 31;24(21):15831. doi: 10.3390/ijms242115831 (PMC10648812; doi:10.3390/ijms242115831)
Supplement: Supplementary file 1 [file ijms-24-15831-s001.zip › Suppl Figure S1.pptx]

## Slide 1
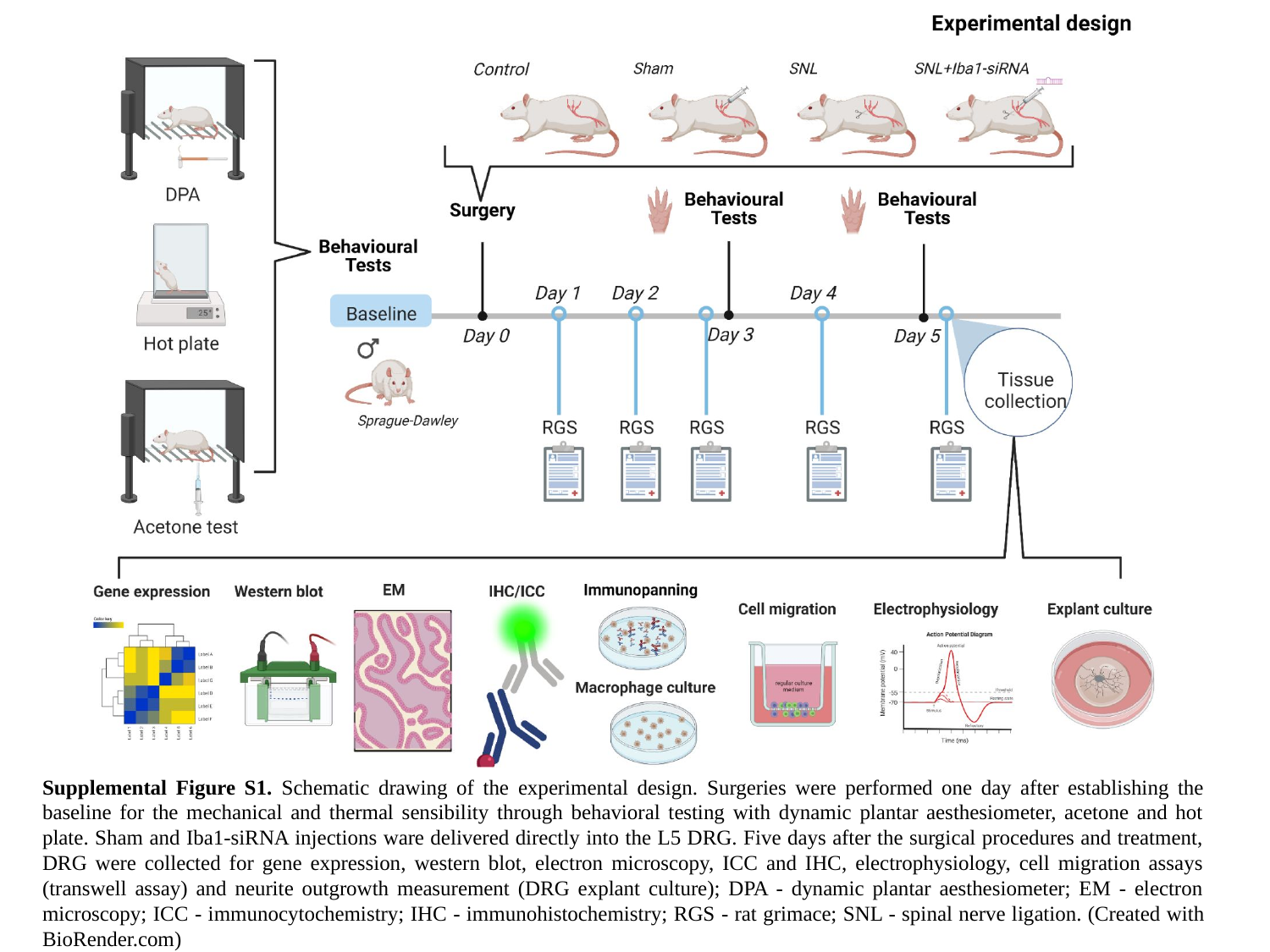

Supplemental Figure S1. Schematic drawing of the experimental design. Surgeries were performed one day after establishing the baseline for the mechanical and thermal sensibility through behavioral testing with dynamic plantar aesthesiometer, acetone and hot plate. Sham and Iba1-siRNA injections ware delivered directly into the L5 DRG. Five days after the surgical procedures and treatment, DRG were collected for gene expression, western blot, electron microscopy, ICC and IHC, electrophysiology, cell migration assays (transwell assay) and neurite outgrowth measurement (DRG explant culture); DPA - dynamic plantar aesthesiometer; EM - electron microscopy; ICC - immunocytochemistry; IHC - immunohistochemistry; RGS - rat grimace; SNL - spinal nerve ligation. (Created with BioRender.com)
