# Supplementary material for: Switching Rat Resident Macrophages from M1 to M2 Phenotype by Iba1 Silencing Has Analgesic Effects in SNL-Induced Neuropathic Pain
Source: Int J Mol Sci. 2023 Oct 31;24(21):15831. doi: 10.3390/ijms242115831 (PMC10648812; doi:10.3390/ijms242115831)
Supplement: Supplementary file 1 [file ijms-24-15831-s001.zip › Suppl Figure S2.pptx]

## Slide 1
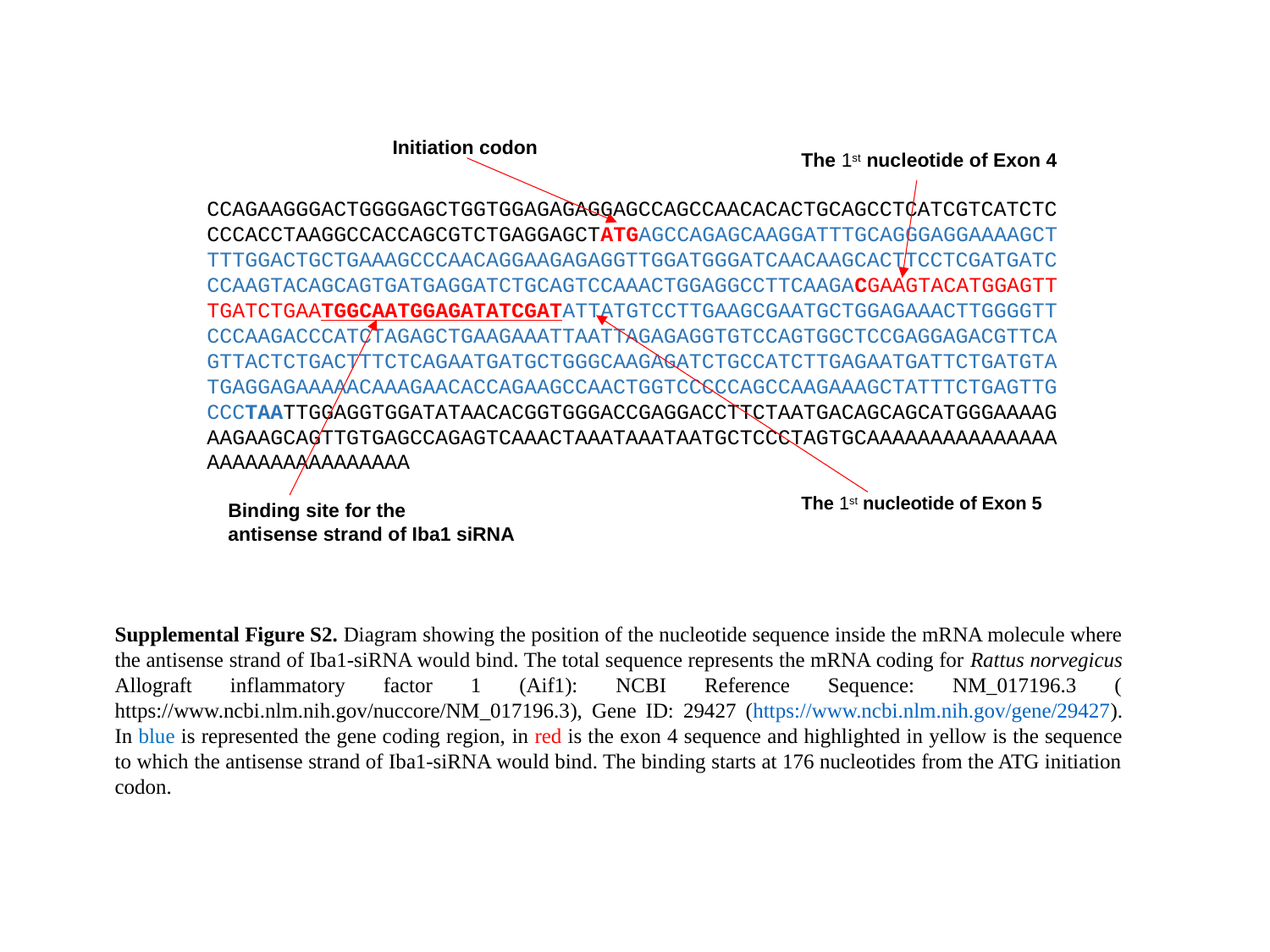

Initiation codon
CCAGAAGGGACTGGGGAGCTGGTGGAGAGAGGAGCCAGCCAACACACTGCAGCCTCATCGTCATCTCCCCACCTAAGGCCACCAGCGTCTGAGGAGCTATGAGCCAGAGCAAGGATTTGCAGGGAGGAAAAGCTTTTGGACTGCTGAAAGCCCAACAGGAAGAGAGGTTGGATGGGATCAACAAGCACTTCCTCGATGATCCCAAGTACAGCAGTGATGAGGATCTGCAGTCCAAACTGGAGGCCTTCAAGACGAAGTACATGGAGTTTGATCTGAATGGCAATGGAGATATCGATATTATGTCCTTGAAGCGAATGCTGGAGAAACTTGGGGTTCCCAAGACCCATCTAGAGCTGAAGAAATTAATTAGAGAGGTGTCCAGTGGCTCCGAGGAGACGTTCAGTTACTCTGACTTTCTCAGAATGATGCTGGGCAAGAGATCTGCCATCTTGAGAATGATTCTGATGTATGAGGAGAAAAACAAAGAACACCAGAAGCCAACTGGTCCCCCAGCCAAGAAAGCTATTTCTGAGTTGCCCTAATTGGAGGTGGATATAACACGGTGGGACCGAGGACCTTCTAATGACAGCAGCATGGGAAAAGAAGAAGCAGTTGTGAGCCAGAGTCAAACTAAATAAATAATGCTCCCTAGTGCAAAAAAAAAAAAAAAAAAAAAAAAAAAAAAA
Binding site for the
antisense strand of Iba1 siRNA
The 1st nucleotide of Exon 4
The 1st nucleotide of Exon 5
Supplemental Figure S2. Diagram showing the position of the nucleotide sequence inside the mRNA molecule where the antisense strand of Iba1-siRNA would bind. The total sequence represents the mRNA coding for Rattus norvegicus Allograft inflammatory factor 1 (Aif1): NCBI Reference Sequence: NM_017196.3 (https://www.ncbi.nlm.nih.gov/nuccore/NM_017196.3), Gene ID: 29427 (https://www.ncbi.nlm.nih.gov/gene/29427). In blue is represented the gene coding region, in red is the exon 4 sequence and highlighted in yellow is the sequence to which the antisense strand of Iba1-siRNA would bind. The binding starts at 176 nucleotides from the ATG initiation codon.
