# Supplementary material for: Switching Rat Resident Macrophages from M1 to M2 Phenotype by Iba1 Silencing Has Analgesic Effects in SNL-Induced Neuropathic Pain
Source: Int J Mol Sci. 2023 Oct 31;24(21):15831. doi: 10.3390/ijms242115831 (PMC10648812; doi:10.3390/ijms242115831)
Supplement: Supplementary file 1 [file ijms-24-15831-s001.zip › Suppl Figure S3.pptx]

## Slide 1
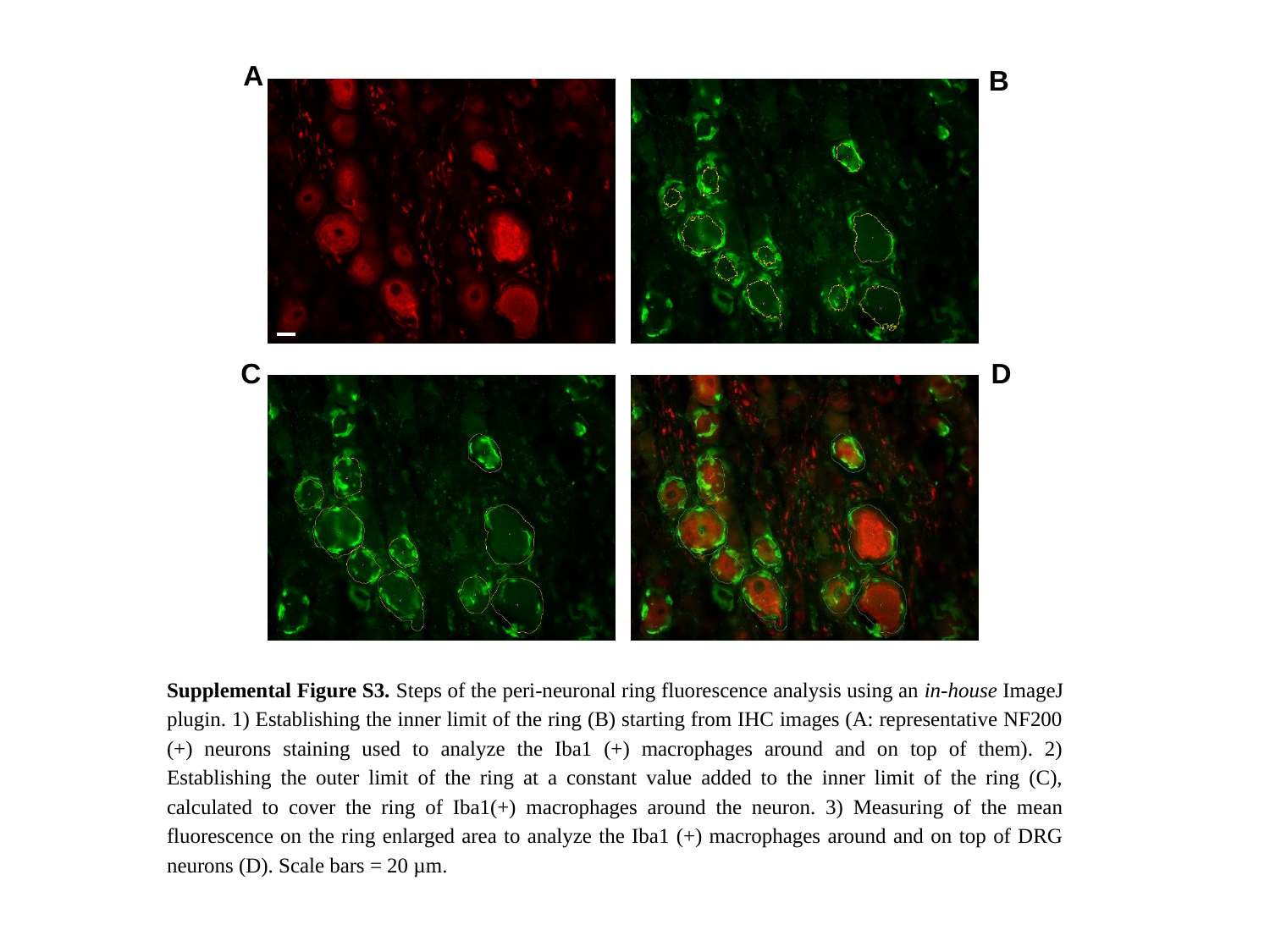

A
B
C
D
Supplemental Figure S3. Steps of the peri-neuronal ring fluorescence analysis using an in-house ImageJ plugin. 1) Establishing the inner limit of the ring (B) starting from IHC images (A: representative NF200 (+) neurons staining used to analyze the Iba1 (+) macrophages around and on top of them). 2) Establishing the outer limit of the ring at a constant value added to the inner limit of the ring (C), calculated to cover the ring of Iba1(+) macrophages around the neuron. 3) Measuring of the mean fluorescence on the ring enlarged area to analyze the Iba1 (+) macrophages around and on top of DRG neurons (D). Scale bars = 20 µm.
