# Supplementary material for: Switching Rat Resident Macrophages from M1 to M2 Phenotype by Iba1 Silencing Has Analgesic Effects in SNL-Induced Neuropathic Pain
Source: Int J Mol Sci. 2023 Oct 31;24(21):15831. doi: 10.3390/ijms242115831 (PMC10648812; doi:10.3390/ijms242115831)
Supplement: Supplementary file 1 [file ijms-24-15831-s001.zip › Suppl Figure S4.pptx]

## Slide 1
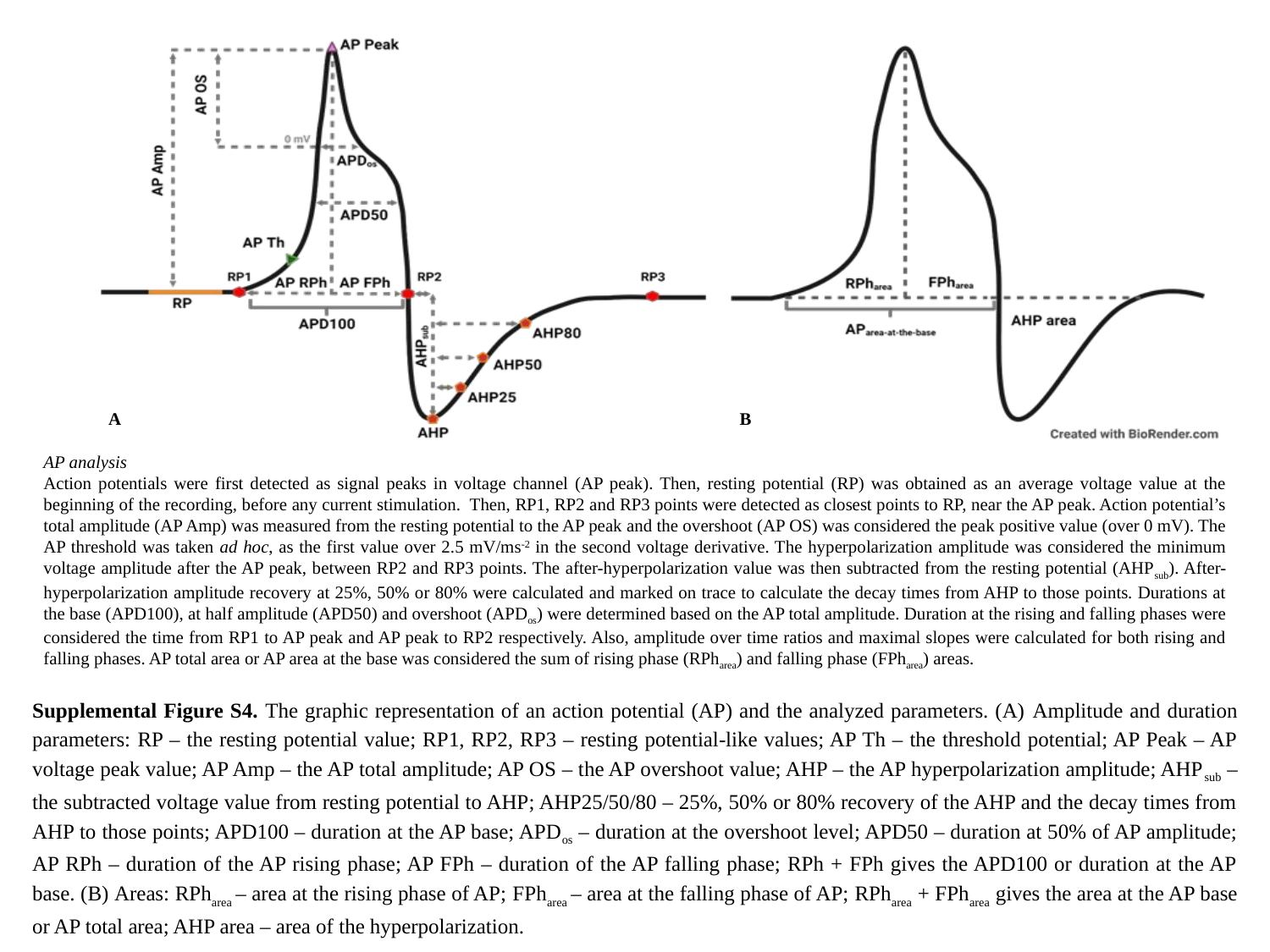

B
A
AP analysis
Action potentials were first detected as signal peaks in voltage channel (AP peak). Then, resting potential (RP) was obtained as an average voltage value at the beginning of the recording, before any current stimulation. Then, RP1, RP2 and RP3 points were detected as closest points to RP, near the AP peak. Action potential’s total amplitude (AP Amp) was measured from the resting potential to the AP peak and the overshoot (AP OS) was considered the peak positive value (over 0 mV). The AP threshold was taken ad hoc, as the first value over 2.5 mV/ms-2 in the second voltage derivative. The hyperpolarization amplitude was considered the minimum voltage amplitude after the AP peak, between RP2 and RP3 points. The after-hyperpolarization value was then subtracted from the resting potential (AHPsub). After-hyperpolarization amplitude recovery at 25%, 50% or 80% were calculated and marked on trace to calculate the decay times from AHP to those points. Durations at the base (APD100), at half amplitude (APD50) and overshoot (APDos) were determined based on the AP total amplitude. Duration at the rising and falling phases were considered the time from RP1 to AP peak and AP peak to RP2 respectively. Also, amplitude over time ratios and maximal slopes were calculated for both rising and falling phases. AP total area or AP area at the base was considered the sum of rising phase (RPharea) and falling phase (FPharea) areas.
Supplemental Figure S4. The graphic representation of an action potential (AP) and the analyzed parameters. (A) Amplitude and duration parameters: RP – the resting potential value; RP1, RP2, RP3 – resting potential-like values; AP Th – the threshold potential; AP Peak – AP voltage peak value; AP Amp – the AP total amplitude; AP OS – the AP overshoot value; AHP – the AP hyperpolarization amplitude; AHPsub – the subtracted voltage value from resting potential to AHP; AHP25/50/80 – 25%, 50% or 80% recovery of the AHP and the decay times from AHP to those points; APD100 – duration at the AP base; APDos – duration at the overshoot level; APD50 – duration at 50% of AP amplitude; AP RPh – duration of the AP rising phase; AP FPh – duration of the AP falling phase; RPh + FPh gives the APD100 or duration at the AP base. (B) Areas: RPharea – area at the rising phase of AP; FPharea – area at the falling phase of AP; RPharea + FPharea gives the area at the AP base or AP total area; AHP area – area of the hyperpolarization.
