# Supplementary material for: Switching Rat Resident Macrophages from M1 to M2 Phenotype by Iba1 Silencing Has Analgesic Effects in SNL-Induced Neuropathic Pain
Source: Int J Mol Sci. 2023 Oct 31;24(21):15831. doi: 10.3390/ijms242115831 (PMC10648812; doi:10.3390/ijms242115831)
Supplement: Supplementary file 1 [file ijms-24-15831-s001.zip › Suppl Figure S5.pptx]

## Slide 1
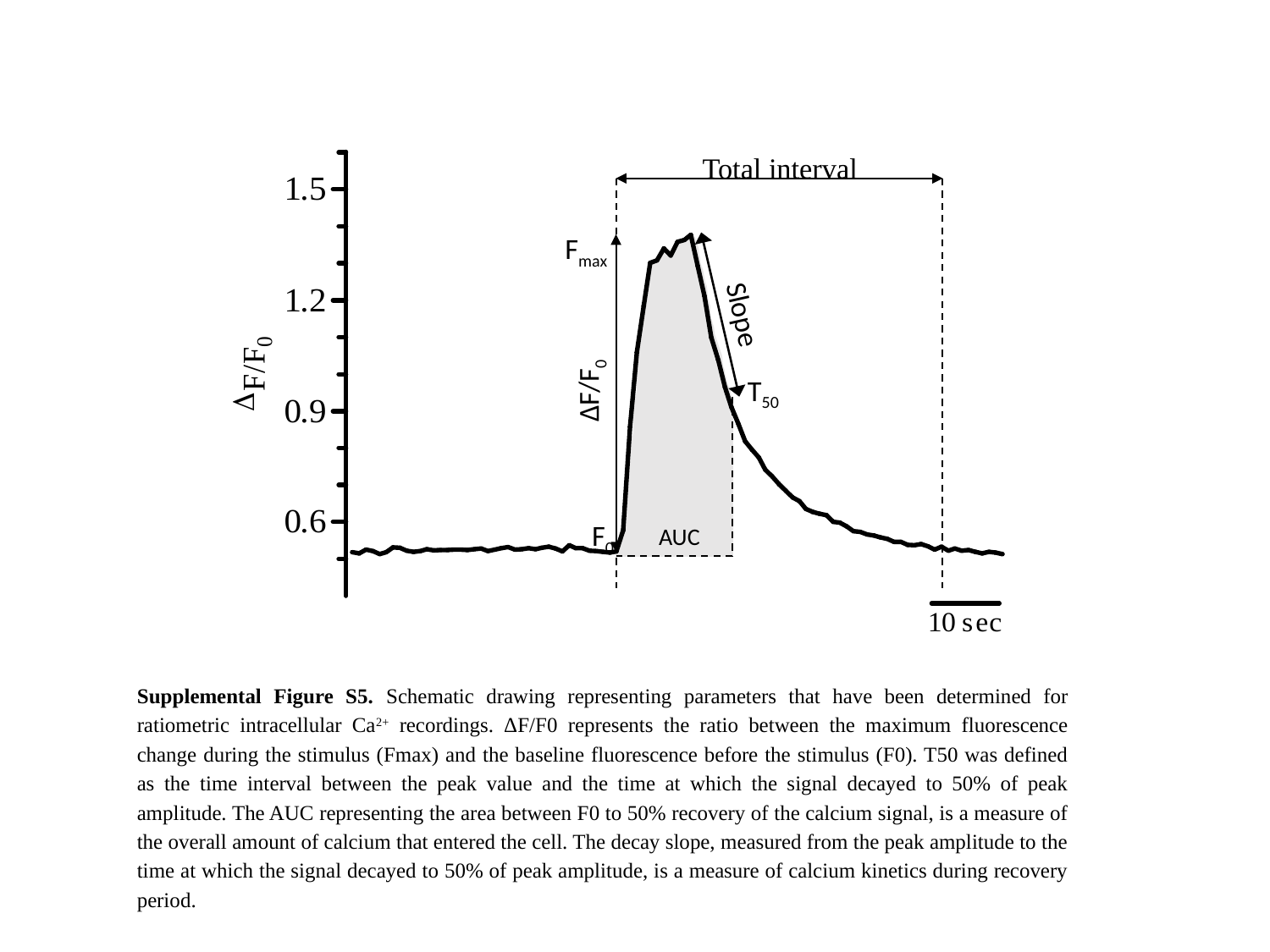

Total interval
Fmax
ΔF/F0
F0
Slope
T50
AUC
Supplemental Figure S5. Schematic drawing representing parameters that have been determined for ratiometric intracellular Ca2+ recordings. ΔF/F0 represents the ratio between the maximum fluorescence change during the stimulus (Fmax) and the baseline fluorescence before the stimulus (F0). T50 was defined as the time interval between the peak value and the time at which the signal decayed to 50% of peak amplitude. The AUC representing the area between F0 to 50% recovery of the calcium signal, is a measure of the overall amount of calcium that entered the cell. The decay slope, measured from the peak amplitude to the time at which the signal decayed to 50% of peak amplitude, is a measure of calcium kinetics during recovery period.
