# Supplementary material for: Switching Rat Resident Macrophages from M1 to M2 Phenotype by Iba1 Silencing Has Analgesic Effects in SNL-Induced Neuropathic Pain
Source: Int J Mol Sci. 2023 Oct 31;24(21):15831. doi: 10.3390/ijms242115831 (PMC10648812; doi:10.3390/ijms242115831)
Supplement: Supplementary file 1 [file ijms-24-15831-s001.zip › Suppl Figure S6.pptx]

## Slide 1
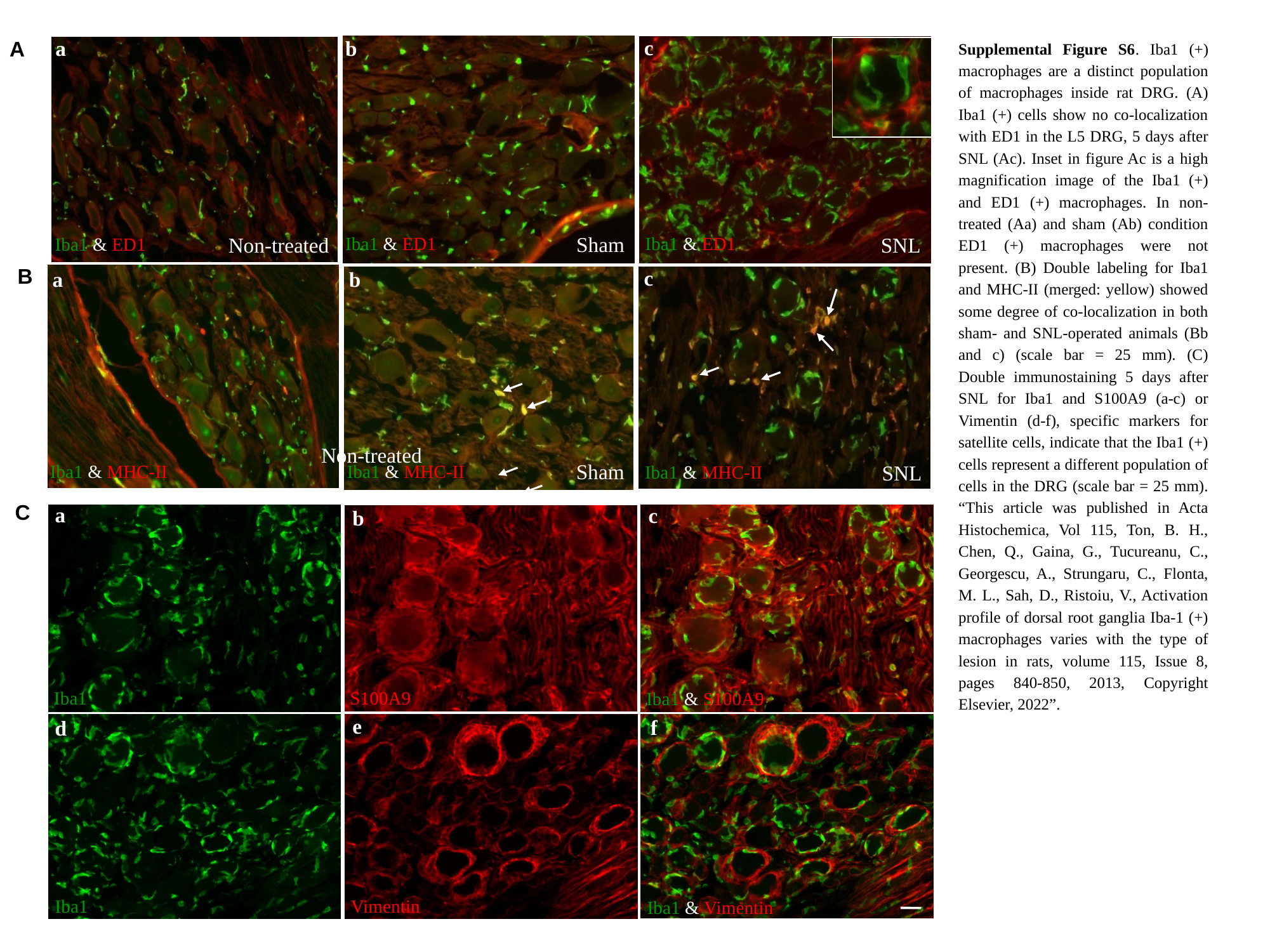

c
a
b
Sham
Non-treated
Iba1 & ED1
SNL
Iba1 & ED1
Iba1 & ED1
c
b
a
Sham
SNL
Iba1 & MHC-II
Iba1 & MHC-II
Iba1 & MHC-II
A
B
C
a
c
b
Iba1
S100A9
Iba1 & S100A9
e
f
d
Iba1
Vimentin
Iba1 & Vimentin
Supplemental Figure S6. Iba1 (+) macrophages are a distinct population of macrophages inside rat DRG. (A) Iba1 (+) cells show no co-localization with ED1 in the L5 DRG, 5 days after SNL (Ac). Inset in figure Ac is a high magnification image of the Iba1 (+) and ED1 (+) macrophages. In non-treated (Aa) and sham (Ab) condition ED1 (+) macrophages were not present. (B) Double labeling for Iba1 and MHC-II (merged: yellow) showed some degree of co-localization in both sham- and SNL-operated animals (Bb and c) (scale bar = 25 mm). (C) Double immunostaining 5 days after SNL for Iba1 and S100A9 (a-c) or Vimentin (d-f), specific markers for satellite cells, indicate that the Iba1 (+) cells represent a different population of cells in the DRG (scale bar = 25 mm). “This article was published in Acta Histochemica, Vol 115, Ton, B. H., Chen, Q., Gaina, G., Tucureanu, C., Georgescu, A., Strungaru, C., Flonta, M. L., Sah, D., Ristoiu, V., Activation profile of dorsal root ganglia Iba-1 (+) macrophages varies with the type of lesion in rats, volume 115, Issue 8, pages 840-850, 2013, Copyright Elsevier, 2022”.
Non-treated
