# Supplementary material for: Switching Rat Resident Macrophages from M1 to M2 Phenotype by Iba1 Silencing Has Analgesic Effects in SNL-Induced Neuropathic Pain
Source: Int J Mol Sci. 2023 Oct 31;24(21):15831. doi: 10.3390/ijms242115831 (PMC10648812; doi:10.3390/ijms242115831)
Supplement: Supplementary file 1 [file ijms-24-15831-s001.zip › Suppl Figure S7.pptx]

## Slide 1
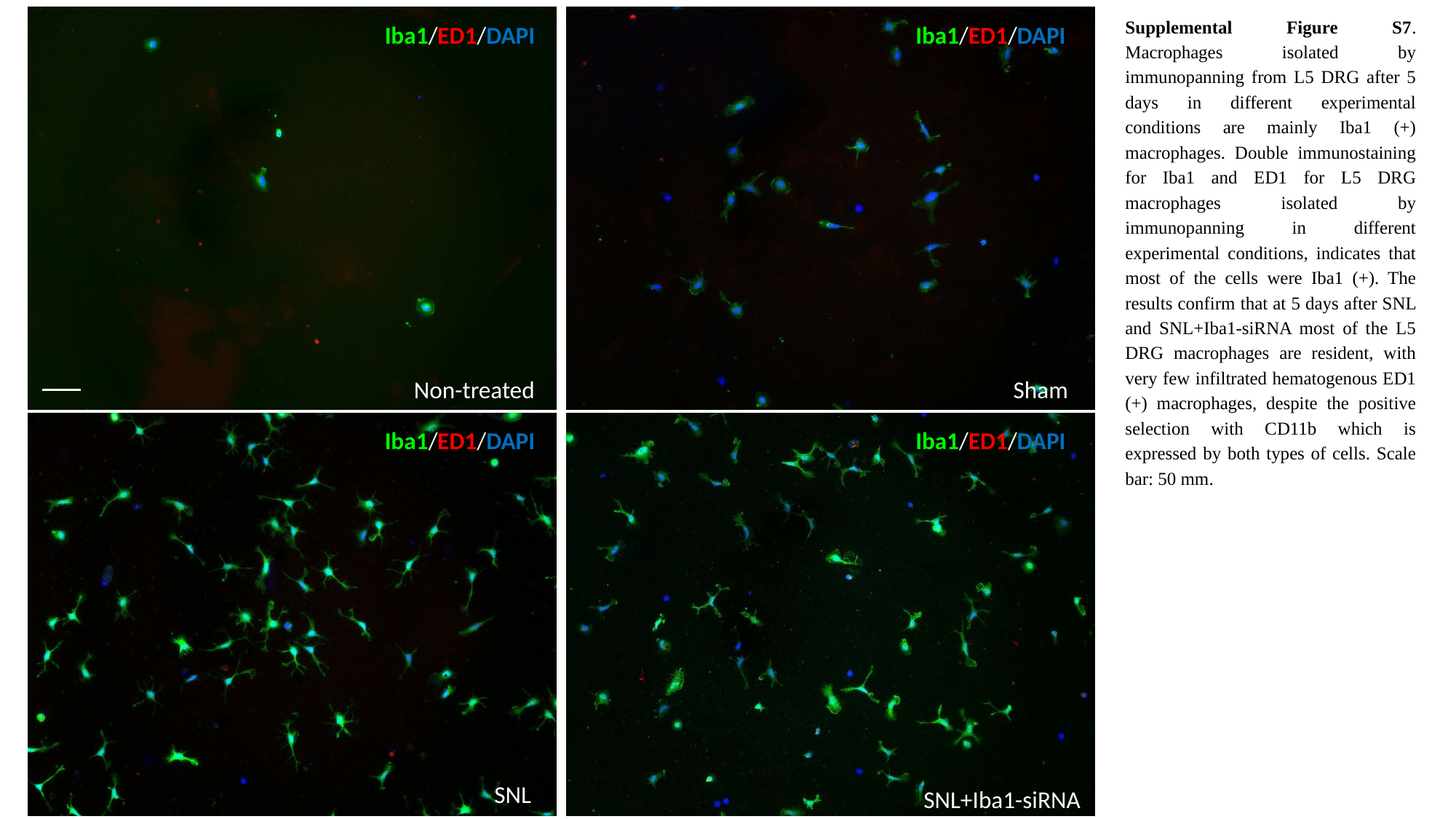

Iba1/ED1/DAPI
Iba1/ED1/DAPI
Non-treated
Sham
Iba1/ED1/DAPI
Iba1/ED1/DAPI
SNL
SNL+Iba1-siRNA
Supplemental Figure S7. Macrophages isolated by immunopanning from L5 DRG after 5 days in different experimental conditions are mainly Iba1 (+) macrophages. Double immunostaining for Iba1 and ED1 for L5 DRG macrophages isolated by immunopanning in different experimental conditions, indicates that most of the cells were Iba1 (+). The results confirm that at 5 days after SNL and SNL+Iba1-siRNA most of the L5 DRG macrophages are resident, with very few infiltrated hematogenous ED1 (+) macrophages, despite the positive selection with CD11b which is expressed by both types of cells. Scale bar: 50 mm.
