# Supplementary material for: Switching Rat Resident Macrophages from M1 to M2 Phenotype by Iba1 Silencing Has Analgesic Effects in SNL-Induced Neuropathic Pain
Source: Int J Mol Sci. 2023 Oct 31;24(21):15831. doi: 10.3390/ijms242115831 (PMC10648812; doi:10.3390/ijms242115831)
Supplement: Supplementary file 1 [file ijms-24-15831-s001.zip › Supplemental Table S1.pdf]

Supplemental Table S1. Forward and reverse sequences of primers used in this study

	Target	Primers' Sequence
**M1 markers**	Rat CD32	F: 5′- GGTGAAACTTGAGCCCCCAT-3′
		R: 5′- TCTGGAGTAGCAGCCAGTCA-3′
	Rat iNOS	F: 5'- CACAGAGGGCTCAAAGGAGG-3'
		R: 5'-AAAGTGGTAGCCACATCCCG -3'
	Rat CD86	F: 5'- CGAACACTATTTGGGCGCAG -3'
		R: 5'- CAAGCCCGTGTCCTTGATCT -3'
**M2 markers**	Rat CD163	F: 5'- CTGGAGCATGAACGAGGTGT-3'
		R: 5'- TTCCTGAGCATCGGTTGTCC-3'
	Rat CD206	F: 5'-CGTTCGCTGATGCAAACCAA -3'
		R: 5'- CCCATAAACCACCTGCCACT-3'
	Rat Arginase-1	F: 5'- GGACATCGTGTACATCGGCT-3'
		R: 5'- GTAGCCGGGGTGAATACTGG-3'
**Pro-inflammatory cytokines**	Rat IL-6	F: 5'-ATATGTTCTCAGGGAGATCTTGGAA-3'
		R: 5'-GTGCATCATCGCTGTTCATACA-3'
	Rat TNF alpha	F: 5'-GCCACCACGCTCTTCTGT-3'
		R: 5'-GGCAGCCTTGTCCCTTGA-3'
	Rat IL-1beta	F: 5'-TCTGTGACTCGTGGGATGAT-3'
		R: 5'-GGCAGCCTTGTCCCTTGA-3'
**Pro-regenerative factors**	Rat BDNF	F: 5'-GGGTGAAACAAAGTGGCTGT-3'
		R: 5'-ATGTTGTCAAACGGCACAAA-3'
	Rat NGF	F: 5'-CCTGCCAGAGTCCTTTTCTG-3'
		R: 5'-GGTTCAGGCCACAAAGTGTT-3'
	Rat neurotrophin-3	F: 5'-GATCCAGGCGGATATCTTGA-3'
		R: 5'-AGCGTCTCTGTTGCCGTAGT -3'
	Rat caspaza-1	F: 5'-CTGGAGCTTCAGTCAGGTCC-3'
		R: 5'-AGGTCAACATCAGCTCCGAC-3'
**Reference genes**	Rat YWHAZ	F: 5'- GAGCCCGTAGGTCATCTTGG-3'
		R: 5'- CCTCAGCCAAGTAGCGGTAG-3'
	Rat 18s	F: 5'- AGTGCCAGCCTCGTCTCATA-3'
		R: 5'- GATGGTGATGGGTTTCCCGT-3'
