# Supplementary material for: Switching Rat Resident Macrophages from M1 to M2 Phenotype by Iba1 Silencing Has Analgesic Effects in SNL-Induced Neuropathic Pain
Source: Int J Mol Sci. 2023 Oct 31;24(21):15831. doi: 10.3390/ijms242115831 (PMC10648812; doi:10.3390/ijms242115831)
Supplement: Supplementary file 1 [file ijms-24-15831-s001.zip › Supplemental Table S2.pdf]

Supplemental Table S2. Protocol for processing the tissue samples for electron microscopy

Protocol Steps	Temperature	Duration
Fixation of the tissue in a 4% glutaraldehyde solution in cacoylate buffer (TCS) 0.1 M, pH 7.3. The tissue was cut in 1 mm^3 pieces.	4 °C	4 -24 h
Washing in TCS 0,1 M	4 °C	2 x 1 h
Post-fixation with OsO_4 0,1 M in TCS	4 °C	1 h
Washing in TCS 0,1 M	4 °C	2x 10-15min
Dehydration in ethanol 30°	4 °C	15-30 min
Dehydration in ethanol 50°	4 °C	15-30 min
Dehydration in ethanol 70°	4 °C	15-30 min
Dehydration in ethanol 90°	4 °C	15-30 min
Dehydration in ethanol 96°	RT	2x15 min
Dehydration in ethanol 100°	RT	3x15 min
Propylene oxide	RT	2x15 min
Bath I: Propylene oxide/Epoxy resin (2/1)	RT	2 h
Bath II: Propylene oxide/Epoxy resin (1/2)	RT	o/n
Bath III: Epoxy resin in open jars (to allow evaporation of propylene oxide)	RT	2-3 h
Inclusion in Epoxy resin in labeled plastic capsules	RT	-
Polymerization of Epoxy resin	60°C	48 h

RT – room temperature; o/n – overnight
