# Supplementary material for: Switching Rat Resident Macrophages from M1 to M2 Phenotype by Iba1 Silencing Has Analgesic Effects in SNL-Induced Neuropathic Pain
Source: Int J Mol Sci. 2023 Oct 31;24(21):15831. doi: 10.3390/ijms242115831 (PMC10648812; doi:10.3390/ijms242115831)
Supplement: Supplementary file 1 [file ijms-24-15831-s001.zip › Supplemental Table S3.pdf]

Supplemental Table S3. Numerical values for behavioral testing (ANOVA repeated measures).

**Experimental group**	**Behavioral tests**	**Baseline**	**Day 3**	**Day 5**
Sham<br>(n = 11)	DPA, threshold	1.00	0.80 ± 0.06<br>▲P = 0. 022	0.72 ± 0.06<br>ϕϕP = 0. 001 and P = 0.645
	DPA, latency	1.00	0.76 ± 0.08<br>▲P = 0. 015	0.65 ± 0.07<br>ϕϕϕP < 0. 001 and P = 0.421
	Acetone	1.00	0.42 ± 0.22<br>P = 0.999	3.2 ± 1.20<br>P = 0.098 and •P =0. 027
	Hot plate	1.00	0.72 ± 0.07<br>▲▲P = 0. 008	0.87 ± 0.05<br>P = 0.381 and P = 0.264
SNL<br>(n = 9)	DPA, threshold	1.00	0.79 ± 0.08<br>▲P = 0. 035	0.45 ± 0.03<br>ϕϕϕP < 0. 001 and •••P < 0. 001
	DPA, latency	1.00	0.75 ± 0.08<br>▲P = 0. 013	0.43 ± 0.04<br>ϕϕϕP < 0. 001 and ••P = 0. 001
	Acetone	1.00	1.6 ± 0.44<br>P = 0.999	7.36 ± 0.94<br>ϕϕϕP < 0. 001 and •••P < 0. 001
	Hot plate	1.00	1.12 ± 0.16<br>P = 0.999	0.63 ± 0.15<br>P = 0.095 and •P = 0. 017
SNL Iba1-siRNA<br>(n =12)	DPA, threshold	1.00	0.73 ± 0.06<br>▲▲P = 0. 008	0.77 ± 0.06<br>ϕP = 0. 028 and P = 0.999
	DPA, latency	1.00	0.73 ± 0.06<br>▲▲P = 0. 009	0.74 ± 0.06<br>ϕP = 0. 012 and P = 0.999
	Acetone	1.00	0.76 ± 0.2<br>P = 0.999	2.24 ± 0.67<br>P = 0.122 and P = 0. 050
	Hot plate	1.00	1.11 ± 0.19<br>P = 0.999	0.76 ± 0.08<br>P = 0.467 and P = 0.109

Legend: ▲P values represent comparisons Day 3 against Baseline, ϕP values represent Day 5 against Baseline; •P values represent comparisons Day 5 against Day 3.
