# Supplementary material for: Switching Rat Resident Macrophages from M1 to M2 Phenotype by Iba1 Silencing Has Analgesic Effects in SNL-Induced Neuropathic Pain
Source: Int J Mol Sci. 2023 Oct 31;24(21):15831. doi: 10.3390/ijms242115831 (PMC10648812; doi:10.3390/ijms242115831)
Supplement: Supplementary file 1 [file ijms-24-15831-s001.zip › Supplemental Table S4.pdf]

Supplemental Table S4. Numerical values for the action potential parameters

Legend: P values (also identified as #P when significant) in the SNL column represent comparisons between SNL and sham condition; P values (also identified as *P when significant) in the SNL+Iba1-siRNA column represent comparisons between SNL+Iba1-siRNA and SNL condition; P values (also identified as ◆*P* when significant) in the SNL+Iba1-siRNA column represent comparisons between SNL+Iba1-siRNA and sham condition

**Target**		**Non treated**	**Sham**	**SNL**	**SNL+Iba1-siRNA**
Resting potential (mV)		-69.460 ± 1.49 (n = 9)	-68.01 ± 2.32 (n = 12)	-61.39 ± 2.22 (n = 13)<br>#P = 0.050	-60.99 ± 2.72 (n = 13)<br>P = 0.910<br>*P = 0.064*
Threshold (mV)		-39.120 ± 1.846 (n = 9)	-38.070 ± 2.199 (n = 12)	-35.310 ± 1.735 (n = 13)<br>P = 0.33	-35.400 ± 2.395 (n = 13)<br>P = 0.976<br>*P = 0.422*
Total amplitude (mV)		126.3 ± 1.92 (n = 9)	117.2 ± 3.42 (n = 12)	106 ± 3.07 (n = 13)<br>#P = 0.022	109 ± 4.99 (n = 12)<br>P = 0.613<br>*P = 0.196*
Overshoot (mV)		57.49 ± 1.95 (n = 9)	49.75 ± 1.94 (n = 12)	45.29 ± 2.49 (n = 13)<br>P = 0.177	48.62 ± 3.53 (n = 13)<br>P = 0.448<br>*P = 0.786*
**AHP amplitude (mV)**	Substracted	13.98 ± 0.84 (n = 9)	14.76 ± 1.08 (n = 12)	12.73 ± 0.52 (n = 13)<br>P = 0.098	11.23 ± 1.24 (n = 13)<br>P = 0.277<br>◆*P = 0.044*
	80% recovery	-75.25 ± 1.35 (n = 9)	-70.97 ± 2.22 (n = 12)	-63.92 ± 2.20 (n = 13)<br>#P = 0.034	-63.23 ± 2.57 (n = 13)<br>P = 0.840<br>◆*P = 0.033*
**Duration (ms)**	at the base	7.51 ± 0.39 (n = 9)	7.57 ± 0.41 (n = 12)	14.03 ± 1.41 (n = 13)<br>###P < 0.001	14.64 ± 1.42 (n = 12)<br>P = 0.763<br>◆◆◆*P < 0.001*

	at 50% amplitude	2.20 ± 0.26 (n = 9)	1.90 ± 0.18 (n = 12)	6.16 ± 1.01 (n = 13)<br>$^{\#\#\#}$P < 0.001	5.86 ± 1.30 (n = 13)<br>P = 0.854<br>◆◆*P = 0.008*
	at overshoot	2.05 ± 0.33 (n = 9)	1.59 ± 0.16 (n = 12)	5.21 ± 1.03 (n = 13)<br>$^{\#\#}$P < 0.003	4.96 ± 1.17 (n = 13)<br>P = 0.876<br>◆*P = 0.012*
	at rising phase	4.26 ± 0.31 (n = 9)	4.83 ± 0.26 (n = 12)	5.31 ± 0.32 (n = 13)<br>P = 0.268	5.91 ± 0.23 (n = 13)<br>P = 0.146<br>◆◆*P = 0.005*
	at falling phase	3.24 ± 0.45 (n = 9)	2.74± 0.24 (n = 12)	8.71 ± 1.23 (n = 13)<br>$^{\#\#\#}$P < 0.001	8.72 ± 1.35 (n = 12)<br>P = 0.996<br>◆◆◆*P < 0.001*
**AHP decay time (ms)**	AHP up to 80% recovery	149.70 ± 20.88 (n = 9)	92.23 ± 15.46 (n = 12)	82.78 ± 11.51 (n = 13)<br>P = 0.625	77.23 ± 13.71 (n = 13)<br>P = 0.759<br>*P = 0.473*
**Area (mV*ms)**	at the base	354.60 ± 26.69 (n = 9)	305.30 ± 14.94 (n = 12)	644.20 ± 82.14 (n = 13)<br>$^{\#\#\#}$P < 0.001	638.1 ± 107.1 (n = 13)<br>P = 0.964<br>◆◆*P = 0.007*
	at rising phase	141.10 ± 10.29 (n = 9)	140.70 ± 8.32 (n = 12)	159.80 ± 13.14 (n = 13)<br>P = 0.240	156.40 ± 12.52 (n = 13)<br>P = 0.853<br>*P = 0.315*
	at falling phase	213.50 ± 31.08 (n = 9)	162.80 ± 13.38 (n = 12)	484.30 ± 75.87 (n = 13)<br>$^{\#\#\#}$P < 0.001	481.70 ± 99.03 (n = 13)<br>P = 0.983<br>◆◆*P = 0.005*
	at AHP	-1433.00 ± 200.5 (n = 9)	-969.9 ± 110.6 (n = 12)	-765.40 ± 93.46 (n = 13)<br>P = 0.169	-688.3 ± 130.1 (n = 13)<br>P = 0.634<br>*P = 0.115*

**Rate** (mV/ms)	Rising phase	31.14 ± 2.70 (n = 9)	25.40 ± 1.93 (n = 12)	21.34 ± 2.23 (n = 13)<br>P = 0.185	18.99 ± 1.32 (n = 13)<br>P = 0.374<br>◆*P = 0.010*
	Falling phase	42.87 ± 3.61 (n = 9)	46.88 ± 4.51 (n = 12)	16.85 ± 3.16 (n = 13)<br>###P < 0.001	15.38 ± 1.88 (n = 13)<br>P = 0.693<br>◆◆◆*P < 0.001*
**Slope (mV)**	Rising phase	17.35 ± 2.58 (n = 9)	7.65 ± 2.75 (n = 12)	5.79 ± 2.95 (n = 13)<br>P = 0.651	4.60 ± 3.12 (n = 13)<br>P = 0.784<br>*P = 0.475*
	Falling phase (mV)	-2.41 ± 7.69 (n = 9)	-7.53 ± 6.36 (n = 12)	-14.62 ± 8.42 (n = 13)<br>P = 0.508	-30.37 ± 9.29 (n = 13)<br>P = 0.217<br>P = 0.058
