# Supplementary material for: Switching Rat Resident Macrophages from M1 to M2 Phenotype by Iba1 Silencing Has Analgesic Effects in SNL-Induced Neuropathic Pain
Source: Int J Mol Sci. 2023 Oct 31;24(21):15831. doi: 10.3390/ijms242115831 (PMC10648812; doi:10.3390/ijms242115831)
Supplement: Supplementary file 1 [file ijms-24-15831-s001.zip › Supplemental Table S5.pdf]

Supplemental Table S5. Numerical values for the levels of mRNA coding for M1 and M2 markers in different experimental conditions

	Target	Non-treated	Sham	SNL	SNL+Iba1-siRNA
**M1 markers**	CD32	1.03 ± 0.08 (n = 3)	9.32 ± 2.10 (n = 3)	11.20 ± 0.80 (n = 3)<br>P = 0.914	5.67 ± 0.90 (n = 3)<br>*P = 0.036<br>*P = 0.199*
	CD86	1.03 ± 0.08 (n = 3)	7.27 ± 0.69 (n = 3)	11.55 ± 1.69 (n = 3)<br>P = 0.093	4.86 ± 1.41 (n = 3)<br>*P = 0.010<br>*P = 0.540*
	iNOS	1.03 ± 0.08 (n = 3)	2.61 ± 0.60 (n = 3)	3.02 ± 0.37 (n = 3)<br>P = 0.999	1.96 ± 0.53 (n = 3)<br>P = 0.403<br>*P = 0.999*
**M2 markers**	CD163	1.03 ± 0.08 (n = 3)	2.74 ± 0.43 (n = 3)	4.37 ± 0.34 (n = 3)<br>$^{\#}$P = 0.012	5.83 ± 0.18 (n = 3)<br>*P = 0.023<br>◆◆◆*P < 0.001*
	Arginase-1	1.03 ± 0.08 (n = 3)	20.25 ± 2.22 (n = 3)	30.78 ± 0.93 (n = 3)<br>P = 0.056	43.55 ± 4.46 (n = 3)<br>*P = 0.022<br>◆◆◆*P < 0.001*
	CD206	1.03 ± 0.08 (n = 3)	2.73 ± 0.50 (n = 3)	5.29 ± 0.89 (n = 3)<br>$^{\#}$P = 0.031	4.43 ± 0.33 (n = 3)<br>P = 0.892<br>*P = 0.174*

Legend: P values (also identified as $^{\#}$P when significant) in the SNL column represent comparisons between SNL and sham condition; P values (also identified as *P when significant) in the SNL+Iba1-siRNA column represent comparisons between SNL+Iba1-siRNA and SNL condition; *P values* (also identified as ◆*P* when significant) in the SNL+Iba1-siRNA column represent comparisons between SNL+Iba1-siRNA and sham condition.
