# Supplementary material for: Switching Rat Resident Macrophages from M1 to M2 Phenotype by Iba1 Silencing Has Analgesic Effects in SNL-Induced Neuropathic Pain
Source: Int J Mol Sci. 2023 Oct 31;24(21):15831. doi: 10.3390/ijms242115831 (PMC10648812; doi:10.3390/ijms242115831)
Supplement: Supplementary file 1 [file ijms-24-15831-s001.zip › Supplemental Table S6.pdf]

Supplemental Table S6. Numerical values for the levels of mRNA coding for pro-inflammatory (Pro-INF) and pro-regenerative (Pro-REG) mediators in different experimental conditions

	Target	Non-treated	Sham	SNL	SNL+Iba1-siRNA
Pro-INF	IL-6	1.00 ± 0.05 (n = 3)	10.89 ± 2.09 (n = 3)	16.18 ± 2.05 (n = 3)<br>P = 0.207	7.04 ± 0.14 (n = 3)<br>*P = 0.026<br>_P = 0.475_
	TNF-α	1.00 ± 0.05 (n = 3)	4.23 ± 0.28 (n = 3)	5.18 ± 0.38 (n = 3)<br>P = 0.306	2.15 ± 0.26 (n = 3)<br>**P = 0.001<br>◆◆_P = 0.008_
	IL-1β	1.00 ± 0.05 (n = 3)	13.99 ± 2.97 (n = 3)	23.49 ± 3.34 (n = 3)<br>P = 0.151	5.71 ± 1.63 (n = 3)<br>*P = 0.011<br>_P = 0.232_
Pro-REG	BDNF	1.00 ± 0.05 (n = 3)	1.38 ± 0.15 (n = 3)	1.08 ± 0.15 (n = 3)<br>P = 0.999	3.67 ± 0.27 (n = 3)<br>***P < 0.001<br>◆◆◆_P < 0.001_
	NGF	1.00 ± 0.05 (n = 3)	2.30 ± 0.28 (n = 3)	1.60 ± 0.10 (n = 3)<br>P = 0.999	10.06 ± 1.40 (n = 11)<br>**P = 0.001<br>◆◆_P = 0.001_
	NT-3	1.00 ± 0.05 (n = 3)	1.65 ± 0.43 (n = 3)	2.23 ± 0.02 (n = 3)<br>P = 0.772	5.49 ± 0.36 (n = 11)<br>**P = 0.001<br>◆◆◆_P < 0.001_

Legend: P values (also identified as #P when significant) in the SNL column represent comparisons between SNL and sham condition; P values (also identified as *P when significant) in the SNL+Iba1-siRNA column represent comparisons between SNL+Iba1-siRNA and SNL condition; *P values* (also identified as ◆*P* when significant) in the SNL+Iba1-siRNA column represent comparisons between SNL+Iba1-siRNA and sham condition.
